# Gold Nanoparticles for Antiviral Applications: Design Principles, Surface Engineering, and Mechanistic Insights

**DOI:** 10.3390/pharmaceutics18070769

**Published:** 2026-06-24

**Authors:** Kang Shu, Yating Lei, Linjie Li, Shike Wang, Ting Du, Ting Tong

**Affiliations:** 1 School of Life and Health Sciences, Hunan University of Science and Technology, Xiangtan 411201, China; shukang@mail.hnust.edu.cn (K.S.); lyt@mail.hnust.edu.cn (Y.L.); llj2025@mail.hnust.edu.cn (L.L.); wsk@mail.hnust.edu.cn (S.W.); 2 State Key Laboratory of Food Nutrition and Safety, College of Food Science and Engineering, Tianjin University of Science and Technology, Tianjin 300457, China; tingdu@tust.edu.cn

**Keywords:** gold nanoparticles, antiviral nanomedicine, surface functionalization, multivalent interactions, viral entry inhibition, photothermal inactivation, vaccine delivery

## Abstract

Gold nanoparticles (AuNPs) have emerged as versatile antiviral nanoplatforms because their size, morphology, plasmonic properties, and surface chemistry can be precisely engineered. In this review, we summarize the core design principles of antiviral AuNPs from a structure–function–mechanism perspective. We first outline representative synthetic and interface-programming routes for AuNP preparation, including citrate reduction, Brust–Schiffrin synthesis, seed-mediated growth, green synthesis, direct thiol-conjugation, and mixed-ligand shell strategies, emphasizing how these approaches define particle size, morphology, surface accessibility, interfacial composition, and downstream biofunctionalization potential. We then discuss major surface engineering strategies, including polyethylene glycol, nucleic acids, antibodies and nanobodies, peptides, glycans, antiviral drugs, and biomimetic coatings, with particular attention to how ligand density, orientation, flexibility, and interfacial stability determine biological performance. Next, we examine how functionalized AuNPs inhibit different stages of the viral life cycle, including viral attachment and entry, intracellular replication, assembly and egress, photothermal inactivation, and immune modulation or vaccine delivery. Finally, we highlight current challenges, including incomplete structure–activity relationships, dynamic nano–bio interactions under physiological conditions, limited standardization across studies, and translational barriers related to safety, reproducibility, and scale-up. This review provides a conceptual framework for the rational development of next-generation AuNP-based antiviral nanotherapeutics.

## 1. Introduction

Viral infections remain a major and evolving threat to global public health, particularly in the context of rapidly mutating RNA viruses, recurrent zoonotic spillover, and the continued emergence of antiviral resistance [1,2]. Although vaccines and direct-acting antiviral drugs have substantially improved the prevention and treatment of many viral diseases, their overall effectiveness can still be limited by antigenic drift, incomplete cross-protection, narrow antiviral spectrum, suboptimal tissue delivery, and the eventual selection of resistant strains [3]. These limitations have stimulated growing interest in antiviral strategies that are mechanistically versatile and adaptable across different viral families.

Nanomaterials have emerged as promising tools in antiviral research because their characteristic dimensions are comparable to those of many virions and subcellular structures involved in infection [4]. This size compatibility enables direct interactions with viral particles, host–cell membranes, and intracellular trafficking pathways. More importantly, nanoparticles can be engineered to integrate multiple functions within a single platform, including blockade of viral attachment, delivery of antiviral cargos, modulation of host–cell responses, and externally triggered inactivation [5]. In contrast to many conventional small-molecule antivirals, which typically act on a single viral or host target, nanoplatforms can be designed to combine recognition, delivery, and therapeutic action at the nano–bio interface.

A wide range of inorganic nanomaterials, including silver [6], copper [7,8], zinc [9,10], selenium [11,12], tellurium [13], iron [14], and silicon [15] systems, have shown antiviral activity through mechanisms such as direct virion disruption, redox-associated damage, adsorption-mediated inhibition, or carrier-enabled delivery of therapeutic agents. Within this broader landscape, AuNPs are especially attractive because they combine controllable synthesis, tunable size and morphology, chemical stability, localized surface plasmonic properties, and well-established surface conjugation chemistry [16]. The gold core provides a structurally robust nanoscale scaffold, while the surface can be readily modified with thiolated polymers, nucleic acids, peptides, proteins, glycans, small-molecule drugs, and biomimetic coatings. As a result, AuNPs can be adapted to distinct antiviral purposes, including multivalent inhibition of virus–host interactions, stabilization and intracellular delivery of labile therapeutics, photothermal inactivation, and enhancement of antiviral immunity [17,18,19,20].

A central concept in AuNP-based antiviral design is multivalency. Many viruses rely on the collective engagement of multiple weak interactions with host–cell receptors, glycans, or attachment factors during the earliest stages of infection. AuNPs provide a nanoscale scaffold capable of presenting ligands at high local density and in controlled spatial arrangements, thereby amplifying binding avidity and enabling biological effects that are difficult to achieve with free ligands alone [21]. In this context, antiviral performance is determined not only by ligand identity, but also by how that ligand is displayed at the nanoparticle interface. Parameters such as ligand density, orientation, linker flexibility, surface charge, and particle curvature can strongly influence colloidal stability, protein adsorption, cellular uptake, intracellular trafficking, and ultimately antiviral outcome.

Over the past decade, increasing evidence has indicated that functionalized AuNPs can inhibit or modulate infection by a wide range of medically relevant viruses, including influenza virus, human immunodeficiency virus (HIV), herpes simplex virus (HSV), respiratory syncytial virus (RSV), hepatitis B virus (HBV), and SARS-CoV-2 [17]. However, the field remains conceptually fragmented. Many studies focus on a specific nanoparticle formulation, a single ligand class, or one antiviral application, whereas fewer analyses systematically connect nanoparticle synthesis, surface engineering, and antiviral mechanism, within a unified design framework. Consequently, it remains difficult to extract generalizable principles that explain how physicochemical design and interfacial architecture jointly determine biological behavior and antiviral efficacy.

In this review, we therefore examine antiviral AuNPs from a structure–function–mechanism perspective. We first summarize the principal synthetic and interface-programming routes used to prepare AuNP cores and functionalizable surfaces for antiviral nanoplatforms, emphasizing how synthesis influences size, morphology, surface accessibility, dispersibility, and ligand-shell design. We then discuss representative surface engineering strategies—including PEGylation, nucleic acid conjugation, antibody and protein coupling, peptide functionalization, glycan display, drug loading, and biomimetic coating—and highlight how these interfacial designs regulate colloidal stability, biological identity, targeting, and cargo handling. Next, we analyze how functionalized AuNPs interfere with different stages of the viral life cycle, including viral attachment and entry, intracellular replication, assembly and egress, photothermal inactivation, and immune modulation or vaccine delivery. Finally, we discuss the major current challenges in antiviral AuNP research, including incomplete understanding of structure–activity relationships, the dynamic complexity of the nano–bio interface, and the lack of standardized comparative frameworks across studies.

## 2. Synthetic Routes for AuNPs Used in Antiviral Nanoplatforms

Gold nanoparticles have been extensively explored as antiviral nanoplatforms because their physicochemical properties can be precisely tuned through synthetic control over core size, morphology, surface charge, ligand density, colloidal stability, and optical response. These parameters are not merely synthetic descriptors; rather, they determine how AuNPs interact with virions, viral envelope proteins, host–cell receptors, endosomal compartments, immune cells, and biological fluids. Therefore, the synthesis of AuNPs for antiviral applications should be viewed as the first layer of antiviral design, preceding subsequent surface engineering and mechanistic optimization.

**Figure 1:**
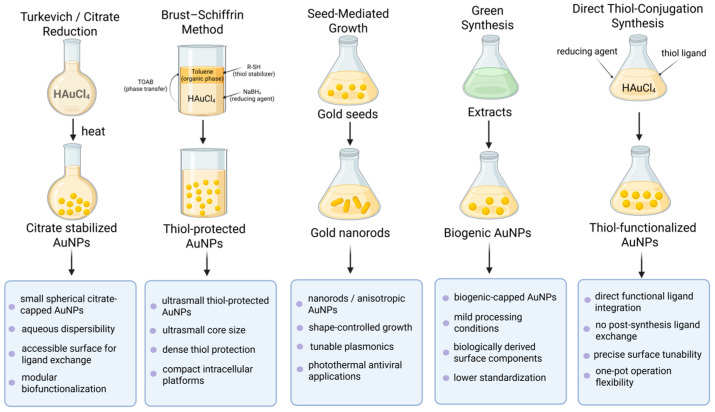
Representative synthetic routes of gold nanoparticles for antiviral nanoplatforms. The diagram highlights five bottom-up approaches: Turkevich Method (Citrate Reduction), Brust–Schiffrin Method, Seed-Mediated Growth, Green Synthesis, and Direct Thiol-Conjugation Synthesis, along with their respective physicochemical properties and application advantages.

In general, AuNP synthesis can be divided into top-down and bottom-up approaches. Top-down methods generate nanoscale gold structures from bulk material through physical processes such as laser ablation, ion sputtering, or mechanical milling. Although these strategies can reduce the use of chemical reducing agents, they often suffer from high energy demand, broader size distributions, limited control over morphology, and less convenient access to aqueous, biomedical-grade dispersions. By contrast, bottom-up synthesis relies on the reduction of soluble gold precursors and offers substantially greater control over nucleation, growth, colloidal stability, and interfacial chemistry [22]. For antiviral and other biomedical applications, bottom-up methods are generally preferred because they are better suited for producing hydrophilic, size-controlled, and readily functionalizable AuNPs [23]. In this section, we focus on representative bottom-up synthetic routes that are most relevant to the rational design of antiviral AuNP systems (Figure 1).

### 2.1. Turkevich Method (Citrate Reduction)

The Turkevich method is one of the most widely used aqueous routes for preparing colloidal AuNPs. In its classical form, chloroauric acid (HAuCl_4_) is reduced by trisodium citrate under boiling conditions, producing citrate-capped AuNPs through coupled nucleation, growth, and stabilization processes. The original study by Turkevich, Stevenson, and Hillier established the fundamental nucleation-and-growth framework for colloidal gold formation, while the subsequent work of Frens demonstrated that particle size could be regulated by controlling the citrate-to-gold precursor ratio [24,25]. In general, higher citrate concentrations favor rapid nucleation and the generation of smaller particles, whereas lower citrate concentrations reduce the number of nuclei and allow more extensive growth, leading to larger AuNPs.

Citrate plays multiple roles in this reaction. It functions as a mild reducing agent, a stabilizing ligand, a pH-modulating component, and an electrostatic capping agent. The resulting AuNPs are typically negatively charged and dispersible in water, making them attractive starting materials for biological and antiviral applications. Their citrate shell is relatively labile, which facilitates subsequent functionalization through ligand exchange with thiolated polyethylene glycol, peptides, carbohydrates, nucleic acids, antibodies, or other biomolecules. This feature explains why citrate-capped AuNPs are frequently used as starting scaffolds for antiviral nanomaterials: the synthesis is simple, inexpensive, scalable at laboratory scale, and compatible with downstream aqueous bioconjugation.

Mechanistic studies have refined the classical view of the Turkevich reaction. Kimling et al. revisited the method and emphasized that particle formation is highly sensitive to precursor concentration, reagent order, heating profile, pH, and reaction time [26]. Tyagi et al. further showed that pH-controlled citrate reduction can produce AuNPs at room temperature, illustrating the importance of protonation state and reduction kinetics in determining nucleation and growth behavior [27]. More recent work has focused on improving the precision and reproducibility of citrate-based synthesis. Dong et al. discussed how optimized control of experimental parameters can yield precision AuNPs with narrower size distributions [28], while Salloum et al. compared citrate-capping approaches and highlighted the importance of optimized wet-chemical protocols for morphology and colloidal stability control [29].

A particularly important recent mechanistic insight comes from real-time surface-enhanced Raman spectroscopy analysis of Turkevich synthesis, which revealed that Au–Cl adlayers modulate the surface chemistry of growing AuNPs [30]. According to this study, the formation, evolution, and collapse of Au–Cl adlayers help explain characteristic optical, electrochemical, and size changes during growth and termination. This observation provides a more unified chemical picture of the Turkevich process: AuNP growth is not governed only by citrate-mediated reduction and electrostatic stabilization, but also by transient halide-containing surface layers that regulate surface reactivity. For antiviral AuNP design, this insight is relevant because residual chloride, citrate coverage, and surface adlayers can influence ligand-exchange efficiency, surface charge, protein adsorption, and subsequent biological interactions.

### 2.2. Brust–Schiffrin Method (Two-Phase Thiolate Method)

The Brust–Schiffrin method is a landmark synthetic strategy for generating small, thiolate-protected AuNPs. Unlike the Turkevich method, which is usually conducted in water and produces citrate-capped particles, the Brust–Schiffrin route typically involves transferring gold precursors from an aqueous phase into an organic phase using a phase-transfer reagent, followed by reduction in the presence of alkanethiols. Although alkanethiols are commonly used in the classical Brust–Schiffrin synthesis, the method is not limited to simple alkanethiol ligands. Aromatic thiols, functionalized thiols, and thiol-terminated, sulfur-containing, or protected thiol precursors such as thioacetates can also be used directly or converted to thiolate-protected AuNPs [31], thereby expanding the accessible ligand shell chemistry and post-synthetic functionalization options. The resulting AuNPs are stabilized by strong Au–S bonds and often possess dense, ordered organic monolayers. This method is particularly important for preparing small AuNPs and gold nanoclusters with controlled organic ligand shells.

Mechanistically, the Brust–Schiffrin synthesis involves several coupled steps, including precursor phase transfer, formation of Au–thiolate intermediates, nucleation, reduction, growth, and monolayer passivation. Perala and Kumar discussed mechanistic aspects of metal nanoparticle formation in this method, emphasizing the relationship between reduction kinetics, thiol concentration, and particle size [32]. Li et al. provided mechanistic insights into two-phase synthesis of organochalcogenate-protected metal nanoparticles, demonstrating the complexity of precursor speciation and interfacial chemistry [33]. Zhu et al. further identified intermediate precursors in the Brust–Schiffrin synthesis, indicating that the reaction pathway proceeds through chemically distinct Au–thiolate species rather than through a simple direct reduction of free gold ions [34]. Halides and phase-transfer components also strongly affect Brust–Schiffrin synthesis. Booth et al. showed that bromide plays a significant role in the formation of thiol-protected AuNPs, influencing precursor chemistry and nanoparticle formation [35]. These findings are important because residual halides or phase-transfer ions can affect particle purification, ligand-shell structure, colloidal stability, and biological compatibility. For antiviral use, such residual components must be carefully removed or controlled, especially when AuNPs are intended for cell-based assays, mucosal administration, or in vivo applications.

The principal advantage of the Brust–Schiffrin method is the formation of robust thiolate-protected AuNPs. The Au–S bond provides high colloidal stability and enables the preparation of particles with hydrophobic, amphiphilic, or functional organic shells. This is useful for constructing antiviral platforms that require dense multivalent ligand presentation, stable drug loading, or controlled interactions with viral envelopes. Thiolate-protected AuNPs can also serve as precursors for ligand exchange, allowing the introduction of PEG, peptides, carbohydrates, or antiviral molecules. In addition, the method is highly relevant to atomically precise gold nanoclusters, whose structure, optical properties, and ligand shells can be controlled at the molecular level [31]. Although most atomically precise nanoclusters have not yet been systematically evaluated as antiviral agents, their well-defined cores and ligand shells may help clarify structure–activity relationships in future antiviral studies.

### 2.3. Seed-Mediated Growth Synthesis Method

Seed-mediated growth is a multistep strategy that enables precise control over the size, morphology, and optical properties of AuNPs. In contrast to one-pot reduction methods, nucleation and growth are separated: small gold seeds are first prepared and then introduced into a growth solution containing a gold precursor, a mild reducing agent, and structure-directing agents such as CTAB, other surfactants, halides, or metal ions. Gold deposition occurs on the preformed seed surface, and particle morphology can be regulated by seed concentration, surfactant composition, additive ions, pH, temperature, and reduction kinetics [36,37,38]. This method is particularly important for producing anisotropic AuNPs, including nanorods, nanostars, nanoprisms, nanocubes, and porous gold particles. Their formation is governed by crystal growth direction, selective ligand adsorption, facet passivation, oxidative etching, and additive-mediated growth modulation [37,38]. Binary surfactant systems can further tune anisotropic growth by modifying micellar organization and surfactant–particle interactions [39].

For antiviral nanoplatforms, the main value of seed-mediated growth lies in its ability to generate AuNPs with shape-dependent plasmonic properties. Compared with spherical particles, anisotropic AuNPs exhibit localized surface plasmon resonance modes and enhanced electromagnetic fields at tips, edges, or porous regions. Gold nanorods are especially attractive because their longitudinal plasmon resonance can be tuned into the NIR region by adjusting aspect ratio, enabling photothermal antiviral strategies based on localized light-induced heating [40,41]. Under appropriate irradiation conditions, such localized heating may contribute to viral envelope disruption, virion inactivation, or modulation of infected-cell responses. Anisotropic morphology may also influence biological interactions beyond optical performance. Surface curvature and geometry affect ligand packing, protein corona formation, membrane contact, and cellular uptake, which can alter AuNP interactions with virions, host–cell membranes, and intracellular compartments. Thus, seed-mediated growth provides a useful synthetic route for designing AuNPs with both optical and biological functionality.

However, the method also has limitations. CTAB and other growth additives may remain on the AuNP surface and cause cytotoxicity or interfere with antiviral assays. Therefore, purification and ligand exchange with biocompatible coatings such as PEG, phospholipids, peptides, zwitterionic ligands, or polysaccharides are usually required. Recent low-CTAB and reproducible two-step seed-mediated protocols have improved the practicality of gold nanorod synthesis for biomedical applications [40,41]. Overall, seed-mediated growth is most suitable when antiviral AuNPs require anisotropic morphology, NIR photothermal response, enhanced optical sensing, or shape-dependent nano–bio interactions.

### 2.4. Green Synthetic Method

Green synthesis refers to the preparation of AuNPs using biological or naturally derived reducing and stabilizing agents, including plant extracts, microbial metabolites, polysaccharides, proteins, amino acids, vitamins, and other phytochemicals. This approach has attracted considerable interest because it can reduce the use of harsh reducing agents, organic solvents, and toxic surfactants. In many green syntheses, biomolecules simultaneously reduce Au(III) to Au(0) and cap the resulting nanoparticles, producing AuNPs with bioorganic surface layers [42,43].

Plant-mediated synthesis typically relies on phytochemicals such as polyphenols, flavonoids, sugars, and sulfur-containing compounds to reduce Au^3+^ to metallic gold and to cap the resulting nanoparticle surface [44]. These reactions are often performed in aqueous media and under comparatively mild temperature and pH conditions. Depending on the composition of the extract, the resulting AuNPs may possess surface-bound natural molecules that contribute to water dispersibility and interfacial compatibility. Microbial synthesis represents another branch of green fabrication, in which bacteria, fungi, or their secreted enzymes mediate gold reduction either intracellularly or extracellularly [45]. In some cases, the resulting particles are associated with biomolecular coatings, including proteins and other metabolites, which can further shape colloidal stability and cellular interactions [46].

For antiviral applications, green synthesis is especially attractive because bioactive capping molecules may contribute to antiviral effects. For example, Meléndez-Villanueva et al. reported virucidal activity of AuNPs synthesized using garlic extract [47]. In a broader surface-chemistry context, gallic-acid-assisted synthesis has yielded monodispersed AuNPs that inhibited HSV attachment and penetration in vitro [48]. In such systems, antiviral performance may arise from a combination of the gold core, nanoparticle size, multivalent surface presentation, and plant-derived organosulfur or phenolic compounds retained on the particle surface. However, in many biogenic systems, the relative contributions of the metallic core, particle size, and retained extract-derived molecules remain difficult to separate, which complicates mechanistic interpretation and standardization. This multifunctional character can be advantageous when the goal is to develop low-cost topical or environmental antiviral agents.

### 2.5. Direct Thiol-Conjugation and Mixed-Ligand Shell Strategies

Direct thiol-capping strategies exploit the strong affinity between sulfur and gold. Sugie et al. reported the generation of AuNPs via direct thiol-capping with THP-protected thiols without prior deprotection, demonstrating that protected thiol chemistry can be used for nanoparticle formation [49]. Lohse et al. developed a direct synthesis of large water-soluble functionalized AuNPs using Bunte salts as ligand precursors, offering a route to water-dispersible particles with controlled functional groups [50]. Such ligand-precursor approaches are useful because they can reduce uncontrolled ligand exchange and produce functional surfaces directly during nucleation and growth.

Recent studies have expanded one-pot functionalization toward biologically relevant ligands. For example, Xu et al. reported one-pot synthesis of functional peptide-modified AuNPs for gene delivery [51], while Ncobeni et al. developed peptide-functionalized PEI-capped AuNPs targeting CD4-positive T lymphocytes [52]. Although these examples are not exclusively antiviral, they illustrate design principles highly relevant to antiviral nanoplatforms: cationic or peptide-bearing AuNPs can bind nucleic acids, promote cellular uptake, or target virus-susceptible immune cells. Similarly, glyco-thiolate capped AuNPs have been developed for biological applications, supporting the use of carbohydrate-functionalized AuNPs to mimic host–cell glycans or block viral lectin interactions [53].

Multidentate ligands and compact hydrophilic shells provide another route to stable biofunctional AuNPs. Oh et al. reported one-pot aqueous growth of biocompatible AuNPs stabilized with bidentate PEG, enabling the synthesis of water-dispersible particles across a broad size range [54]. Temur et al. used dihydrolipoic acid ligands under photoirradiation to assist AuNP synthesis, benefiting from ethanol oxidation and bidentate thiol binding [55]. Susumu et al. further described thioctic acid-based compact hydrophilic ligands for biocompatible quantum dots and AuNPs, emphasizing the utility of multidentate anchoring for stable biological interfaces [56]. These ligand systems are important for antiviral applications because multidentate or bidentate anchoring can reduce ligand desorption, improve colloidal stability in biological media, and preserve functional ligand display during viral or cellular interactions.

Mixed-ligand shell strategies are particularly powerful for antiviral AuNPs. In such systems, two or more ligands are co-displayed on the nanoparticle surface to balance stability, targeting, immune modulation, and antiviral activity. For example, PEG can provide steric stabilization and reduce nonspecific protein adsorption, while peptides, glycans, nucleic acids, antibodies, or small-molecule antivirals provide specific biological function. The ability to control the ratio between inert stabilizing ligands and active antiviral ligands is essential for optimizing multivalent binding without causing aggregation or cytotoxicity. Borsley et al. recently reported a general one-step synthesis of alkanethiyl-stabilized AuNPs with control over core size and monolayer functionality, demonstrating the feasibility of simultaneously tuning particle core and ligand-shell composition [57].

Because each synthetic route generates AuNPs with distinct core sizes, morphologies, surface states, and functionalization compatibility, the choice of synthesis method should be aligned with the intended antiviral mechanism. The major synthetic routes discussed above and their relevance to antiviral AuNP design are summarized in Table 1.

### 2.6. Summary of Synthetic Considerations for Antiviral Design

As summarized in Table 1, each synthetic route offers a distinct balance among core-size control, morphology regulation, surface accessibility, and downstream functionalization compatibility. For antiviral applications, the choice of AuNP synthesis method should therefore be regarded as a design decision rather than a purely preparative one. Because antiviral efficacy often depends on multivalent ligand presentation, intracellular accessibility, colloidal stability, or photothermal responsiveness, the synthetic route should be selected in alignment with the intended mechanism of action.

The choice of AuNP synthetic route strongly influences downstream antiviral performance. Citrate reduction is ideal for producing aqueous, negatively charged, readily functionalized spherical AuNPs, but these particles often require additional stabilization before biological use. Brust–Schiffrin synthesis provides small, thiolate-protected AuNPs with robust organic monolayers, but organic solvents and hydrophobic ligands may complicate biomedical translation. Seed-mediated growth is indispensable for anisotropic AuNPs with tunable plasmonic and photothermal properties, yet surfactant residues and ligand exchange remain key challenges. Green synthesis offers sustainable and potentially bioactive AuNPs, although reproducibility and mechanistic clarity require improvement. Direct ligand-assisted and mixed-ligand strategies enable more integrated preparation of functional antiviral AuNPs by combining synthetic stabilization with biological function.

For antiviral nanoplatforms, several synthetic parameters should be prioritized. First, particle size must be matched to the intended mechanism: smaller particles may favor cellular uptake and intracellular delivery, whereas larger or multivalent particles may more effectively block viral attachment. Second, morphology should be selected according to optical and biological requirements; spherical particles are often preferred for reproducible conjugation, whereas rods, stars, or other anisotropic structures are advantageous for photothermal inactivation or shape-dependent virus binding. Third, surface chemistry must be compatible with biological media. Residual citrate, CTAB, halides, phase-transfer agents, or plant-derived compounds can affect cytotoxicity and antiviral readouts. Fourth, ligand density and spatial organization should be controlled because antiviral inhibition often relies on multivalent interactions with viral surface proteins or host–cell receptors. Finally, synthetic reproducibility and purification are essential for translating AuNP antiviral systems from proof-of-concept studies to standardized preclinical evaluation.

In practice, no single synthetic method is universally optimal. Instead, synthesis must be considered together with the target virus, intended antiviral mechanism, administration route, safety requirements, and downstream functionalization strategy. A rational antiviral AuNP platform therefore begins not with surface modification alone, but with deliberate selection of a core synthesis route that is compatible with the desired interfacial architecture and biological role. In this sense, synthesis defines the design space within which functionalized AuNPs can be engineered for antiviral performance.

## 3. Surface Engineering Strategies for Antiviral AuNPs

Although the optical and physicochemical properties of AuNPs are strongly influenced by their core size and morphology, their biological identity is determined primarily at the interface. Surface engineering governs colloidal stability, protein adsorption, immune recognition, biodistribution, cellular uptake, cargo loading, and target specificity. In antiviral systems, the interfacial architecture, rather than the gold core alone, largely determines whether AuNPs act as passive carriers, multivalent entry inhibitors, intracellular delivery vehicles, photothermal agents, or immune-modulating platforms.

The importance of surface engineering is especially evident in biological environments, where nanoparticles rapidly encounter salts, proteins, membranes, and cellular barriers that can alter their colloidal behavior and biological fate. A ligand shell that is effective in buffer may perform very differently in serum-containing media or in vivo. Accordingly, antiviral performance depends not only on the identity, but also on ligand density, orientation, flexibility, anchoring stability, hydrodynamic size, and resistance to nonspecific protein corona formation. These interfacial parameters determine whether functionalized AuNPs retain colloidal integrity, preserve access to their active ligands, and engage viral or host targets with sufficient selectivity and avidity.

**Figure 2:**
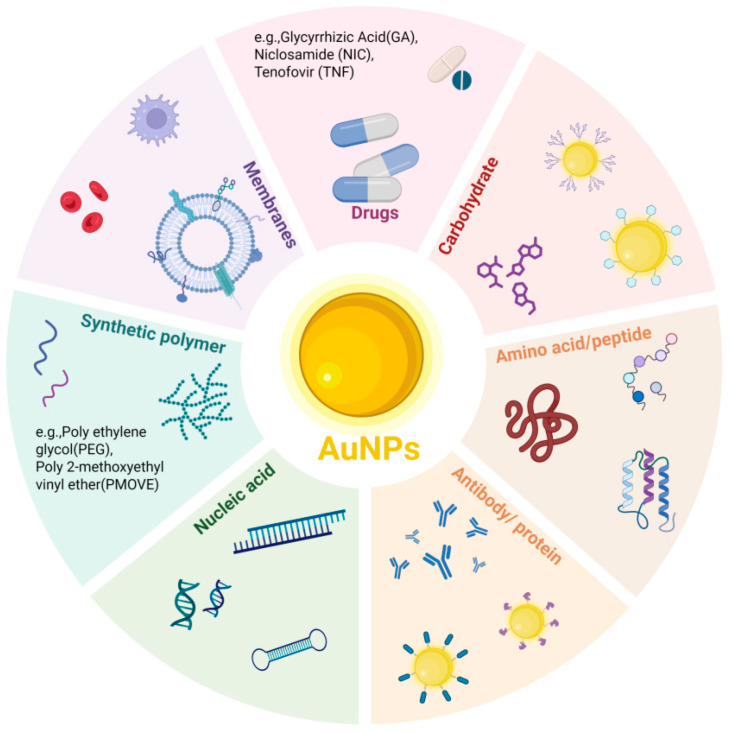
Schematic overview of the major surface functionalization strategies of AuNPs for antiviral applications.

A wide range of surface functionalization strategies has therefore been explored in antiviral AuNP design. PEGylation and related hydrophilic coatings are used primarily to stabilize nanoparticles and improve pharmacological behavior. Nucleic acids, antibodies, nanobodies, peptides, and glycans provide targeting, multivalent recognition, or intracellular regulatory activity. Additional strategies use AuNPs as carriers for antiviral drugs or cloak them with biomimetic coatings to improve biological compatibility and interface control. In practice, effective antiviral AuNPs often combine several of these elements within a single construct. The major functionalization categories discussed in this section are schematically summarized in Figure 2.

### 3.1. Polyethylene Glycol (PEG) Coating

PEGylation is one of the most widely used strategies for improving the colloidal stability and in vivo compatibility of AuNPs. Thiol-terminated polyethylene glycol (PEG) chains can be anchored to the gold surface through Au–S bonds, generating a hydrated steric barrier that reduces nonspecific protein adsorption, suppresses aggregation, and prolongs blood circulation by decreasing rapid clearance by the mononuclear phagocyte system [60]. The performance of PEG coatings depends on parameters such as molecular weight, grafting density, and chain conformation, all of which influence steric stabilization and resistance to protein corona formation.

In antiviral nanoplatforms, PEG is typically not the active antiviral component. Instead, its main role is to preserve colloidal integrity in physiological media, improve pharmacokinetic behavior, and create a biocompatible interfacial background for further incorporation of bioactive ligands such as peptides, aptamers, oligonucleotides, or drugs [61]. Thus, PEG should be viewed primarily as an enabling interfacial component rather than as a direct antiviral ligand.

An additional advantage of PEGylation is its compatibility with mixed-ligand surface designs. PEG can be combined with targeting or recognition motifs to produce nanoparticles that retain serum stability while acquiring selective interactions with viral or cellular targets [62]. In this context, PEG functions as an interfacial spacer and stealth component that helps preserve the accessibility of more active ligands. However, PEG is not completely inert. Incomplete stealth behavior, possible interference with ligand accessibility at high grafting densities, residual protein adsorption, and concern over anti-PEG immune responses have prompted the exploration of alternative hydrophilic coatings, including zwitterionic polymers and poly(2-methoxyethyl vinyl ether) [63,64]. Even so, PEGylation remains a foundational strategy in antiviral AuNP design, particularly for formulations intended for systemic administration. However, repeated administration may induce or be affected by anti-PEG antibodies and accelerated blood clearance, which should be considered for prophylactic or repeated antiviral dosing.

### 3.2. Nucleic Acid Conjugation

Nucleic acid functionalization is among the most versatile strategies for engineering antiviral AuNPs. Oligonucleotides can be immobilized on gold surfaces through thiol-mediated Au-S anchoring, yielding well-defined nanostructures capable of gene regulation, molecular recognition, and therapeutic delivery [65]. Non-covalent approaches, including electrostatic adsorption, hydrogen bonding, and base–gold interactions, can also be used to assemble nucleic acids onto AuNPs without prior chemical modification [66,67]. A major advantage of non-covalent loading is tunable and reversible cargo association, which can be exploited in stimuli-responsive release systems controlled by pH, ionic strength, or other environmental cues [68].

In antiviral applications, nucleic acids can serve as small-interfering RNA (siRNA), antisense agents, targeting aptamers, or structural components in higher-order architectures such as spherical nucleic acids (SNAs) [69,70]. One major application of nucleic acid functionalization is the delivery of gene-silencing cargos. Conjugation to AuNPs can protect siRNA from nuclease degradation, enhance cellular uptake, and improve intracellular delivery relative to free oligonucleotides. For example, AuNP–siRNA constructs targeting dengue virus genes inhibited viral replication in vitro, while ultrasmall AuNPs carrying siRNA directed against both herpes simplex virus and pro-inflammatory NF-κB signaling achieved simultaneous antiviral and anti-inflammatory effects [71,72]. Polyvalent display of siRNA on AuNP surfaces may further improve gene-silencing efficiency by increasing local nucleic acid density and facilitating cellular engagement [73].

A second important role of nucleic acid functionalization is molecular targeting. Aptamers conjugated to AuNPs can bind viral proteins or infected-cell-associated targets with high affinity and selectivity, thereby enabling direct antiviral inhibition, targeted delivery, or both [69,74]. More broadly, densely arranged oligonucleotide shells, as exemplified by SNAs, impart distinctive biological behaviors that differ from those of free linear nucleic acids, including altered cellular uptake profiles and enhanced resistance to enzymatic degradation [70]. These properties make nucleic acid-functionalized AuNPs attractive for antiviral applications that require protected cargo delivery, programmable recognition, and intracellular activity.

### 3.3. Antibody, Nanobody, and Protein Functionalization

Protein attachment can be achieved through non-covalent adsorption, hydrophobic interactions, Au–S coordination via cysteine residues, or covalent coupling through preinstalled reactive groups such as carboxyl or amine functionalities [75]. Non-covalent adsorption is experimentally simple and can preserve protein activity under optimized conditions, but protein orientation and conformational stability remain sensitive to pH, ionic strength, competing proteins, and nanoparticle surface chemistry [76,77]. Covalent strategies generally provide greater attachment stability, but random cross-linking may compromise antigen-binding activity unless site-specific or orientation-controlled conjugation is used. Therefore, successful protein functionalization requires careful optimization of orientation, surface density, structural integrity, and binding stability.

In antiviral applications, protein-functionalized AuNPs are especially useful for enhancing binding avidity through multivalent presentation. For example, nanobodies targeting the SARS-CoV-2 spike protein exhibited markedly enhanced neutralizing potency when displayed on an AuNP scaffold, illustrating how nanoparticle-mediated spatial organization can amplify otherwise monovalent binding interactions [78]. More broadly, antibody- or protein-modified AuNPs can be designed to recognize viral envelope proteins, infected-cell markers, or other disease-relevant molecular targets, thereby supporting selective antiviral action at the nano–bio interface.

Proteins can also serve general interfacial roles beyond direct targeting. Adsorbed protein layers, such as albumin coatings, may improve colloidal stability under challenging environmental conditions and reduce aggregation [79]. However, intentional protein functionalization should be distinguished from uncontrolled protein corona formation. Once AuNPs enter serum-containing media or in vivo environments, adsorbed biomolecules can mask engineered ligands, alter biodistribution, change immune recognition, and redirect cellular uptake [80].

### 3.4. Amino Acid and Peptide Conjugation

Amino acids and peptides are versatile ligands for AuNP functionalization because they offer synthetic accessibility, structural programmability, and biologically meaningful recognition motifs. In some cases, amino acids can participate directly in AuNP synthesis as reducing or stabilizing agents [81]. More commonly, peptides are introduced as post-synthetic surface ligands to provide receptor targeting, membrane interaction, intracellular trafficking, or direct antiviral recognition.

Peptide conjugation is typically achieved through terminal cysteine residues or other anchoring groups capable of establishing stable interactions with the gold surface. Because peptide sequence can be precisely designed, peptide-functionalized AuNPs can be tailored to mimic protein interaction domains, bind host–cell receptors, facilitate cellular uptake, or engage viral surface proteins [62,82]. This sequence-level programmability enables rational adjustment of charge distribution, hydrophobicity, secondary-structure propensity, and receptor affinity. In antiviral settings, multivalent peptide display has been used to enhance inhibition of viral fusion, as illustrated by HIV fusion-inhibitory peptide-functionalized AuNPs [83].

The relevance of peptide functionalization to antiviral AuNP design lies in this combination of biological specificity and nanoscale adaptability. Peptides are smaller and synthetically more accessible than full proteins or antibodies, which reduces steric burden and allows greater freedom for tuning ligand density and surface organization. However, peptide activity is highly sensitive to interfacial context. Surface density, orientation, linker composition, and local crowding can all affect peptide accessibility and function, and the same sequence may behave differently when free in solution versus immobilized on a nanoparticle surface [84]. Peptide conjugation should therefore be regarded as an interfacial design problem requiring careful optimization of both sequence and presentation.

### 3.5. Carbohydrate (Glycan) Conjugation

Glycan functionalization is particularly important in antiviral AuNP design because many viruses initiate infection by recognizing carbohydrates on host–cell surfaces. Decorating AuNPs with monosaccharides, oligosaccharides, polysaccharides, or glycomimetic ligands enables the construction of multivalent glyconanoparticles that mimic host receptors or engage viral lectin-like proteins with enhanced avidity [85,86]. From a synthetic perspective, glycans can be conjugated to AuNPs through several distinct strategies. Thiol-modified carbohydrates can be directly anchored to the gold surface via strong Au–S bonds, either during nanoparticle synthesis or through post-synthetic ligand exchange. Alternatively, glycans can be attached to pre-functionalized AuNP scaffolds using covalent coupling chemistries, such as EDC/NHS reactions with amine-derivatized sugars or click chemistry. Furthermore, one-pot green synthesis approaches allow polysaccharides to act simultaneously as both reducing and stabilizing agents, directly yielding glycan-coated AuNPs. These strategies exploit the nanoscale architecture of AuNPs to achieve dense and spatially organized glycan presentation [87].

The antiviral value of glycan conjugation lies primarily in multivalent binding. Compared with monovalent sugars, glycan-displaying AuNPs can exhibit markedly enhanced affinity toward lectins, viral surface proteins, or receptor-binding interfaces because multiple weak interactions are engaged cooperatively [88]. This principle is illustrated by low-mannose-functionalized glyconanoparticles, which showed enhanced binding affinity to Cyanovirin-N variants relative to the corresponding monomeric ligands [88]. Such findings underscore a central point in antiviral glyconanotechnology: biological performance depends not only on glycan identity, but also on nanoscale presentation.

In practical antiviral design, a broad range of glycans and glycan-derived materials has been explored. Naturally derived polysaccharides such as chitosan and alginate are widely used because of their biocompatibility, ease of modification, and ability to support stable glycosylated AuNP formulations [89]. Smaller carbohydrate motifs, including mannose-, mannuronic-acid-, and sialic-acid-containing ligands, are also of particular interest because they are directly relevant to viral attachment pathways [90,91]. Since many viruses depend on glycan-mediated recognition during the earliest stages of infection, glycan-functionalized AuNPs offer a rational route to competitive inhibition of virus–host interactions through receptor mimicry and multivalent avidity.

Glycan-related or glycoside-containing surface chemistries can also incorporate naturally derived bioactive molecules. For example, glycyrrhizic acid, a triterpenoid saponin glycoside, has been used to functionalize AuNPs; the resulting glycyrrhizic acid-modified AuNPs exhibited inhibitory activity against porcine reproductive and respiratory syndrome virus and SARS-CoV-2 pseudovirus in vitro [92]. Although these systems may not function through identical mechanisms, they illustrate how carbohydrate-rich or polyphenol-associated surface chemistries can contribute to antiviral activity at the stage of viral attachment and entry.

At the same time, the effectiveness of glycan-functionalized AuNPs is governed by more than glycan composition alone. Ligand density, spatial arrangement, linker flexibility, surface curvature, and colloidal stability all influence whether displayed glycans can productively engage viral targets [85,86,90]. Because glycan–protein interactions are often individually weak, insufficient control over nanoscale presentation can markedly reduce antiviral performance.

### 3.6. Drug Loading and Intracellular Delivery

AuNPs can also be engineered as carriers for antiviral drugs through covalent conjugation, electrostatic association, hydrophobic loading, or surface-assisted encapsulation. In these systems, surface functionalization is designed not only to attach the therapeutic cargo, but also to regulate release behavior, improve pharmacokinetics, enhance accumulation at target cells or tissues, and reduce off-target toxicity. Because the AuNP surface can be co-engineered with targeting ligands, protective polymers, or stimuli-responsive components, drug-loaded AuNPs are best viewed as programmable delivery interfaces rather than passive carriers.

Among these strategies, covalent drug attachment offers stable loading and can support sustained release when biodegradable or otherwise cleavable linkers are used. A representative example is tenofovir (TNF) tethered to AuNPs through biodegradable linkages, which enabled prolonged release and demonstrated improved antiviral performance in an in vivo HIV-related proof-of-concept model [93]. This type of design is relevant when the parent drug suffers from short residence time, poor intracellular retention, or suboptimal tissue exposure.

Non-covalent loading approaches can also be valuable, particularly for drugs whose activity benefits from triggered release or co-presentation with recognition elements. Drug molecules may associate with AuNPs through ionic interactions, van der Waals forces, hydrophobic interactions, or nucleic-acid-mediated encapsulation, allowing release to be influenced by local pH or other stimuli, although premature release and nonspecific adsorption remain important design concerns [94,95]. For example, aptamer-functionalized AuNPs have been developed as targeted delivery systems in which molecular recognition is coupled to selective drug release at the target site [96]. In such platforms, therapeutic performance depends not only on the loaded drug, but also on the architecture of the outer surface, which governs colloidal stability, target engagement, and release conditions.

From an antiviral nanomedicine perspective, drug-loaded AuNPs are especially attractive when free drugs are limited by poor solubility, rapid degradation, insufficient intracellular access, or dose-limiting systemic exposure. However, their success depends on careful optimization of drug loading efficiency, release kinetics, colloidal stability, and target selectivity. AuNP-based drug delivery should therefore be understood as an integrated interfacial design problem in which delivery chemistry must be aligned with antiviral mechanism and biological context [97].

### 3.7. Biomimetic Coating

Biomimetic coating represents an emerging surface engineering strategy in which AuNPs are cloaked with natural biological membranes or membrane-inspired materials to impart functions that are difficult to achieve with conventional synthetic ligands alone [98]. These functions may include prolonged circulation, immune evasion, homotypic targeting, and biologically relevant interfacial signaling [99,100]. Depending on the intended application, the coating material can be derived from erythrocytes, immune cells, cancer cells, or other cell-associated membrane sources, each conferring distinct biological properties.

For antiviral applications, biomimetic coating is conceptually attractive because viruses themselves exploit membrane-level recognition, adhesion, and trafficking processes. By decorating AuNPs with cell-derived membrane components, it may be possible to prolong residence time, enhance compatibility in vivo, target inflamed or infected tissues, or create decoy-like interfaces that intercept virus–host interactions [101,102]. Although direct examples of biomimetic-coated AuNPs for antiviral therapy remain relatively limited, related membrane-coated antiviral nanoplatforms provide important design principles for AuNP engineering. For example, Aich et al. recently reported biomimetic copper nanoparticles coated with ACE2-overexpressing cell membranes for selective SARS-CoV-2 neutralization and disinfection, demonstrating that receptor-enriched membrane cloaking can create virus-trapping decoy interfaces with antiviral and environmental decontamination potential [8]. In this sense, biomimetic functionalization extends antiviral design beyond single-ligand recognition and toward more complex control of the nano–bio interface.

However, the biological complexity that makes biomimetic strategies attractive also makes them technically demanding. Membrane source selection, coating integrity, compositional heterogeneity, storage stability, and large-scale reproducibility all remain substantial challenges [99,100]. At present, biomimetic coating should therefore be regarded as a promising frontier rather than a standardized solution in antiviral AuNP engineering. Its greatest potential lies in applications where complex interface control, prolonged biological compatibility, or decoy-like recognition is needed.

### 3.8. Summary of Surface Engineering Principles

Taken together, the functionalization strategies discussed above illustrate that the antiviral utility of AuNPs depends less on the gold core alone than on the structure and function of the interfacial layer. PEGylation primarily improves colloidal stability and pharmacokinetic behavior; nucleic acids provide programmability, intracellular regulatory activity, and molecular recognition; antibodies, nanobodies, and proteins enable highly specific targeting; peptides offer modular recognition with sequence-level tunability; glycans support multivalent receptor mimicry and lectin engagement; drug-oriented functionalization enables controlled delivery; and biomimetic coatings introduce complex biological interface properties. Each strategy contributes a distinct set of capabilities, but none operates independently of the broader physicochemical context established by the nanoparticle surface.

When designing surface engineering strategies for spherical AuNPs intended for use in colloidal suspensions, the final hydrodynamic size is a critical parameter. The attachment of polymers (e.g., PEG), peptides, or targeting antibodies inevitably increases the hydrodynamic diameter of the bare nanoparticles. While a larger hydrodynamic size provided by these bulky coatings ensures excellent steric stabilization and prevents aggregation in complex biological fluids, an excessively large ligand shell may introduce steric hindrance. This hindrance can shield the active targeting groups or reduce the binding efficiency between the AuNPs and the viral surface proteins. Therefore, precisely tuning the ligand length, molecular weight, and grafting density is essential to achieve an optimal hydrodynamic size that balances colloidal stability with maximum antiviral efficacy.

In the case of anisotropic AuNPs, the role of shape must be carefully considered during surface engineering. The distinct crystallographic facets of anisotropic structures (such as the tips versus the longitudinal sides of gold nanorods) often exhibit different binding affinities for capping agents, which can be exploited for site-specific or asymmetric functionalization. Furthermore, the shape directly dictates the geometric matching and contact area with the virus. For instance, rod-like, star-shaped, or spike-bearing nanoparticles can provide larger contact areas or multiple binding points (multivalent interactions) compared to spherical counterparts, allowing them to better match the curvature of the viral envelope and significantly enhance the binding affinity and subsequent viral neutralization.

A recurring principle across these approaches is that ligand identity alone is not sufficient to predict biological performance. Ligand density, orientation, linker flexibility, anchoring stability, colloidal robustness, and resistance to protein corona formation can all determine whether a given functional motif remains accessible and effective under biologically relevant conditions. As summarized in Table 2, different coatings show distinct capacities to reduce, reshape, or tolerate protein adsorption. PEGylated, zwitterionic, and mixed-charge surfaces are generally used to reduce nonspecific fouling, whereas charged, nucleic acid-, peptide-, protein-, and glycan-based surfaces often provide essential antiviral functions but require additional optimization to preserve ligand accessibility in serum-containing or mucosal media.

Another important interfacial parameter is the final hydrodynamic size of the functionalized AuNP. Hydrodynamic diameter integrates the gold core, ligand shell, hydration layer, and adsorbed biomolecules, and therefore often better reflects the biologically relevant size than the metallic core diameter alone. For spherical AuNPs, hydrodynamic diameter can strongly influence colloidal stability, cellular uptake, renal filtration, tissue penetration, mononuclear phagocyte system clearance, and biodistribution. For anisotropic AuNPs, however, additional descriptors such as aspect ratio, effective hydrodynamic dimension, surface curvature, and ligand distribution should also be considered, because a single average diameter cannot fully capture shape-dependent biological behavior.

Surface engineering should therefore be regarded as the central bridge connecting nanoparticle synthesis to antiviral mechanism. It is through this interfacial layer that core physicochemical features are translated into biological identity and therapeutic function. A rational understanding of antiviral AuNPs must consequently focus not only on what ligands are present, but also on how they are organized, stabilized, and presented in complex biological environments. This interfacial perspective provides the foundation for understanding the antiviral mechanisms discussed in the following section.

## 4. Antiviral Mechanisms of Functionalized AuNPs

The antiviral effects of AuNPs arise from the coordinated interplay among nanoscale architecture, surface chemistry, and biological context rather than from the metallic core alone. Core size and morphology influence optical behavior, cellular interactions, and biodistribution, whereas surface functionalization governs colloidal stability, molecular recognition, cargo loading, and interactions with biological interfaces. Functionalized AuNPs should therefore be understood as programmable antiviral nanoplatforms whose activity is engineered at the nano–bio interface.

Depending on their design, functionalized AuNPs can intervene at multiple stages of the viral life cycle. Some systems act at the earliest stage of infection by blocking viral attachment and entry through receptor mimicry, multivalent engagement, steric shielding, or direct virucidal interactions. Others function after internalization by delivering antiviral cargos, modulating host–cell signaling, or altering intracellular conditions required for viral replication. Additional formulations interfere with virion assembly, budding, or release, whereas plasmonically active AuNPs can inactivate virions or infected cells through photothermal effects. AuNPs have also been developed as vaccine carriers, adjuvants, and immunomodulatory platforms that enhance antiviral host responses.

These mechanisms should not be regarded as mutually exclusive. A single AuNP formulation may combine several functions simultaneously, such as serum-stable circulation, multivalent viral capture, intracellular cargo delivery, and immune activation. Conversely, apparently similar nanoparticle systems may behave very differently if they differ in size, shape, ligand density, interfacial flexibility, or susceptibility to protein corona formation. The mechanisms discussed below should therefore be interpreted through a structure–function lens.

### 4.1. Polyvalent Blockade of Viral Attachment and Cellular Entry

Viral attachment and entry are attractive intervention points because they represent the earliest and often rate-limiting steps of infection. Functionalized AuNPs are particularly well suited for targeting this stage because their surfaces can display ligands at high local density and in controlled spatial arrangements, thereby generating multivalent interactions that are typically much stronger than those achieved by the corresponding monovalent molecules. As a result, AuNPs can function either as synthetic mimics of host–cell receptors or as multivalent antagonists of viral attachment and fusion proteins.

The antiviral behavior of entry-targeting AuNPs is closely related to the chemical nature of their surface ligands. Anionic sulfate, sulfonate, and carboxylate groups can mimic glycosaminoglycans or other negatively charged attachment factors and interfere with electrostatic interactions between viral envelope proteins and cell-surface receptors. Carbohydrate- and glycan-derived ligands, such as sialic acid, mannose derivatives, and mannuronic acid, can compete with host–cell glycans or engage viral lectin-like domains. Protein-, peptide-, aptamer-, antibody-, and nanobody-functionalized AuNPs generally provide more sequence- or structure-specific recognition of viral surface proteins, including spike, hemagglutinin, and fusion proteins. These representative relationships between surface chemistry and entry-stage antiviral mechanisms are summarized in Table 3.

One major design principle is the mimicry of host attachment factors. Heparan sulfate proteoglycans (HSPGs), for example, are used by many enveloped viruses during initial attachment. Therefore, sulfate- or sulfonate-terminated AuNPs can act as synthetic HSPG analogues that competitively inhibit viral binding, as demonstrated for HIV-1 and HSV-1 [18,19]. In sulfate-ended AuNPs, antiviral activity is strongly influenced by ligand density, supporting the importance of multivalent HSPG-like presentation [18]. Against HSV-1, mercaptoethane sulfonate-capped AuNPs inhibited viral attachment, entry, and cell-to-cell spread, indicating that densely displayed anionic sulfonate groups can block multiple early infection events [19]. Similar logic applies to other glycan-dependent entry pathways. Sialic-acid-functionalized AuNPs can compete with host–cell sialylated receptors for influenza hemagglutinin binding, thereby suppressing viral attachment and infection in a size-dependent manner [20].

A second major strategy is the direct multivalent engagement of viral glycans or envelope proteins. Polyvalent nano-lectin systems and glycomimetic AuNPs have shown antiviral activity against SARS-CoV-2 by targeting glycans on the viral spike protein [108,109,110]. These studies demonstrate that antiviral potency depends not only on ligand identity, but also on ligand density, orientation, flexibility, and nanoparticle size relative to the viral surface. Beyond glycan-targeting strategies, AuNPs displaying multivalent peptide fusion inhibitors can suppress HIV-1 membrane fusion [83], while galectin-1-conjugated AuNPs strengthen glycan-mediated recognition of influenza virus through clustered carbohydrate-binding motifs [111]. Similarly, polyvalent nanobody architectures organized on AuNP scaffolds improve SARS-CoV-2 neutralization by optimizing spatial engagement of the spike protein [78]. Adaptable self-assembled monolayer shells can further enhance multivalent viral capture by allowing lateral ligand mobility at the nanoparticle interface [112].

In addition to reversible entry blockade, some AuNP systems exert direct virucidal effects by inducing irreversible structural damage to virions. In certain systems, multivalent binding simply prevents productive interaction with host cells, whereas in others the nanoparticle-virus interaction appears to generate mechanical or structural stress that compromises the viral envelope or surface proteins. For example, poly(styrene sulfonate)-coated AuNPs shifted the antiviral action of linear poly(styrene sulfonate) from a predominantly reversible virustatic mechanism toward an irreversible virucidal mechanism [113]. Mixed-charge AuNPs can also preserve antiviral performance more effectively than single-ligand sulfonated particles under high-protein conditions, highlighting the importance of interfacial robustness in complex biological environments [105]. More broadly, virucidal nanoparticle design has shown that flexible sulfonated ligands can generate sufficient multivalent binding forces to deform enveloped virions irreversibly, providing a useful conceptual framework for interpreting related AuNP-based systems [58].

**Figure 3:**
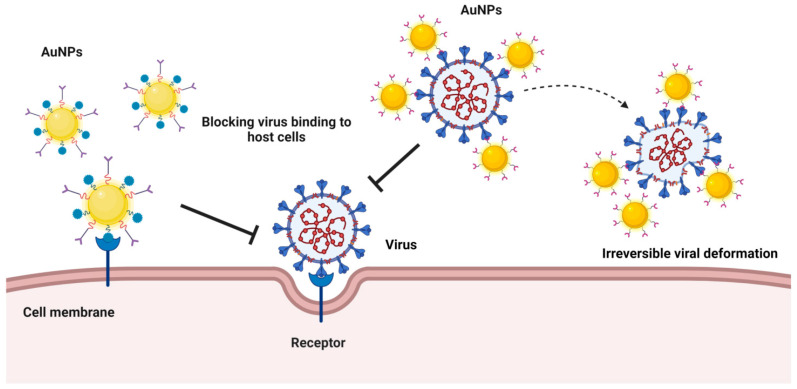
Schematic illustration of the antiviral mechanism of gold nanoparticles (AuNPs). Functionalized AuNPs interact with viral surface proteins, thereby blocking virus binding to host–cell receptors on the cell membrane. The dashed arrow indicates that multivalent binding of AuNPs to the viral envelope/capsid can induce structural disruption and irreversible viral deformation, ultimately inhibiting viral entry and infectivity.

Additional entry-stage mechanisms have also been described. Mannuronic-acid-coated AuNPs and related glycan-targeting platforms can interfere with glycan-mediated interactions on enveloped viruses [91], whereas porous gold nanoparticles have been shown to bind disulfide-rich viral glycoproteins such as influenza hemagglutinin and attenuate their fusogenic function [114]. Bioactive surface ligands may also contribute to entry inhibition, as illustrated by quercetin-conjugated or gallic-acid-associated AuNPs that suppress early virus–host interactions [48,115]. Collectively, these examples indicate that AuNP-mediated entry inhibition is governed by a combination of multivalent avidity, interfacial geometry, functional-group chemistry, and, in some cases, the capacity to physically or conformationally disrupt the virion (Figure 3). The illustrated processes represent mechanistic categories and may not occur simultaneously in all AuNP formulations.

**Table 3:** Representative relationships between AuNP surface chemistry and entry-stage antiviral mechanisms.

Surface Chemistry or Ligand Class	Preferential Binding Mode	Main Target Interface	Representative Viruses	Mechanistic Implication	References
Sulfate or sulfonate groups	HSPG-like electrostatic mimicry and multivalent binding	Glycosaminoglycan-dependent attachment interfaces	HIV-1, HSV-1, HSV-2, HSPG-utilizing enveloped viruses	Inhibits viral attachment and entry, wherein dense or flexible anionic ligands can induce irreversible virucidal structural deformation	[18,19,58,113]
Carboxylate or uronic-acid-containing glycans	Glycan-like recognition and competitive receptor blockade	Glycan-binding viral proteins or lectin-related attachment pathways	EBOVpp	Supports receptor mimicry and enables multivalent inhibition of glycan-mediated viral attachment	[91]
Sialic acid ligands	Host receptor mimicry coupled with multivalent hemagglutinin binding	Hemagglutinin–sialylated receptor interaction	Influenza virus	Competitively blocks host–cell sialylated receptors, with the antiviral efficacy being highly dependent on nanoparticle size and ligand presentation	[20]
Mannose/glycomimetic ligands and lectin domains	Multivalent lectin–glycan recognition	Viral envelope glycans, including spike-associated glycans	SARS-CoV-2-related systems, influenza virus	Translates weak monomeric lectin–glycan interactions into high-avidity viral capture and potent neutralization	[83,108,109,110,111]
Peptide fusion inhibitors	Multivalent presentation of entry co-receptor antagonists	Host CCR5 co-receptor	HIV-1	Multivalent presentation on the nanoparticle surface significantly enhances otherwise weak fusion-inhibitory activity	[83]
Antibodies, nanobodies, and aptamers	Structure- or sequence-specific molecular recognition	Viral surface antigens or infected-cell markers	SARS-CoV-2, cytomegalovirus, rabies-virus-related systems	Enhances binding avidity and enables targeted viral neutralization, cargo delivery, or photothermal therapy	[69,78,96,116,117]
Porous or disulfide-reactive gold surfaces	Direct interaction with disulfide-rich viral glycoproteins	Hemagglutinin and other fusogenic glycoproteins	Influenza A virus	Attenuates viral fusogenic function through the structural perturbation of viral envelope glycoproteins	[114]

Taken together, these studies show that the entry stage is one of the clearest settings in which the nanoscale advantages of AuNPs can be exploited. Their antiviral efficacy at this step arises not simply from ligand presence, but from controlled ligand presentation, interfacial adaptability, and the ability to convert weak molecular recognition into high-avidity capture or even irreversible virucidal action. Notably, most mechanistic insights in this area still derive from in vitro studies, and systematic evaluation of protein corona effects, mucosal environments, in vivo biodistribution, and potential viral escape remains limited. Despite these translational gaps, viral attachment and entry blockade remains one of the most mature and mechanistically informative applications of functionalized AuNPs in antiviral nanomedicine.

### 4.2. Intracellular Disruption of Viral Replication

Once viruses have entered host cells, antiviral intervention becomes more challenging because therapeutic agents must reach intracellular sites of replication while retaining stability, bioactivity, and sufficient local concentration. Functionalized AuNPs provide a versatile platform for this stage because they can protect labile cargos, enhance cellular uptake, and integrate targeting, delivery, and intracellular action within a single nanoscale construct. In contrast to entry inhibitors, intracellular antiviral AuNPs must negotiate endocytic uptake, vesicular trafficking, cargo release, and, in some cases, endosomal escape.

**Figure 4:**
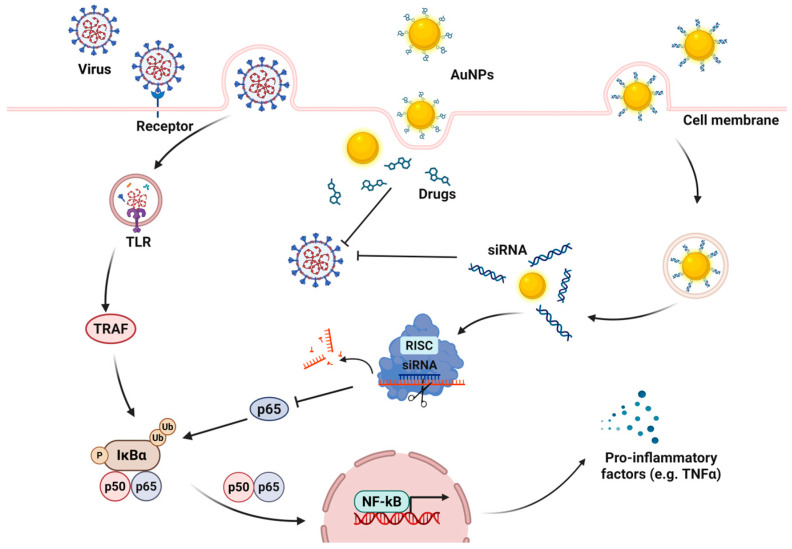
Schematic illustration of the multifunctional antiviral mechanisms of AuNPs. AuNPs can inhibit viral infection through multiple pathways, including direct interference with virus–host cell interactions, delivery of antiviral drugs, and transport of small interfering RNA (siRNA). Following cellular uptake, siRNA-loaded AuNPs promote RNA interference via the RNA-induced silencing complex (RISC), thereby suppressing viral gene expression. In addition, AuNPs modulate host immune signaling pathways, including the TLR/TRAF/NF-κB axis, leading to reduced activation of pro-inflammatory mediators such as TNFα. These pathways represent representative mechanisms reported for different AuNP formulations rather than simultaneous actions of a single platform.

One major application is the delivery of nucleic acid therapeutics. As described in Figure 4, AuNP-conjugated small-interfering RNA (siRNA) can be shielded from nuclease degradation while benefiting from improved cellular internalization relative to free oligonucleotides. This principle has been demonstrated in antiviral systems targeting dengue virus, where AuNP-bound siRNA retained activity even after RNase exposure, whereas free siRNA lost efficacy under comparable conditions [71]. Similarly, ultrasmall AuNPs conjugated with siRNA directed against both herpes simplex virus and pro-inflammatory NF-κB signaling simultaneously suppressed viral replication and inflammatory signaling, illustrating how a single intracellular nanoplatform can combine antiviral and host-modulatory functions [72].

AuNPs have also been developed as carriers for small-molecule antiviral agents, particularly when free drugs are limited by poor pharmacokinetics, insufficient intracellular access, or off-target exposure. Conjugation of tenofovir to AuNPs improved antiviral performance and enabled sustained release in vivo, highlighting the potential of AuNPs to extend the activity window of established antivirals [93]. More advanced formulations combine cargo delivery with molecular targeting; for example, aptamer-conjugated, hyaluronic-acid-coated AuNPs designed for pH-responsive niclosamide delivery to SARS-CoV-2-infected cells illustrate how selective accumulation and controlled intracellular release can be integrated within one nanoscale platform [96]. These systems show that AuNPs can modulate when, where, and under what intracellular conditions antiviral agents become active.

Beyond serving as delivery vehicles, AuNPs may influence intracellular host–cell environments in ways that suppress viral propagation. Studies comparing ligand-stabilized gold nanoclusters, including glutathione-, histidine-, and 2-mercaptoethane sulfonate-coated formulations, have shown that subtle differences in surface chemistry can produce markedly different effects on the proliferation of pseudorabies virus and porcine reproductive and respiratory syndrome virus [118,119]. These observations highlight that intracellular antiviral behavior depends not only on the therapeutic payload, but also on how the nanoparticle surface reshapes the local cellular environment.

Emerging platforms extend this concept toward programmable intracellular antiviral systems. A notable example is AuNP-mediated delivery of CRISPR-Cas13d machinery for targeted cleavage of viral RNA after cellular uptake, representing a modular strategy for antiviral intervention at the level of nucleic acid recognition and degradation [120]. In addition, even relatively simple citrate-capped AuNPs have been reported to reduce foot-and-mouth disease virus replication primarily when administered after infection, suggesting that post-entry antiviral effects may occur even without highly elaborate surface engineering, although the contribution of nonspecific cellular stress or assay-dependent effects should be carefully excluded [121]. However, in such systems the precise intracellular mechanisms are less clearly resolved and should be interpreted cautiously.

Taken together, the intracellular antiviral value of AuNPs lies in two complementary capabilities: protected delivery of therapeutic cargos and modulation of host–cell environments relevant to viral replication. At the same time, successful intracellular antiviral design requires more than nanoparticle uptake alone. Endosomal escape, controlled subcellular trafficking, timely cargo release, and selective accumulation in infected cells remain major challenges. Accordingly, the promise of functionalized AuNPs in this context depends on continued progress in aligning interfacial design with the intracellular biology of viral infection.

### 4.3. Interference with Late-Stage Viral Assembly and Egress

In addition to blocking entry or suppressing intracellular replication, functionalized AuNPs can interfere with later stages of the viral life cycle, including assembly, budding, and release. These mechanisms are particularly relevant because they limit the production and dissemination of infectious progeny rather than merely preventing the initial round of infection. Compared with entry inhibition, however, late-stage antiviral effects are often more difficult to assign to a single molecular event, since they may arise from combined perturbation of membrane organization, intracellular trafficking, organelle function, and viral particle maturation.

**Figure 5:**
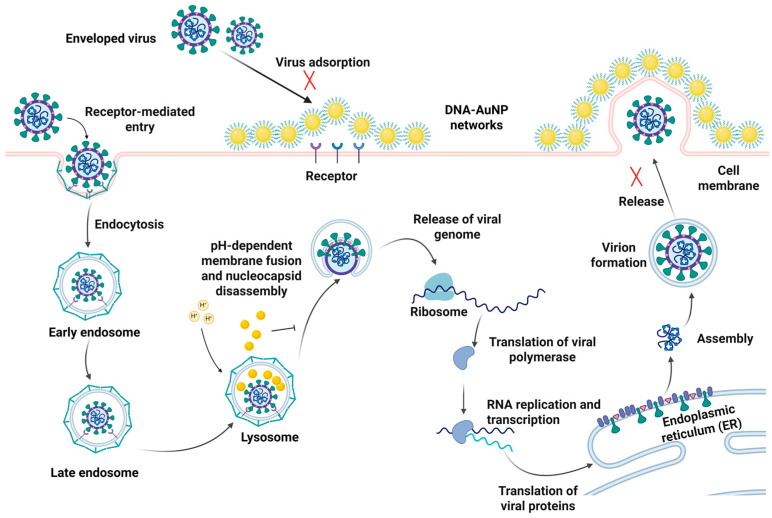
Schematic illustration of the antiviral mechanisms of DNA-conjugated gold nanoparticles (DNA-AuNP) against enveloped viruses. DNA–AuNP networks formed on the cell membrane can act as physical barriers to inhibit viral adsorption to host-cell receptors and interfere with the release of progeny virions during viral budding. In addition, internalized gold nanoparticles can accumulate in lysosomes and induce lysosomal alkalinization, thereby preventing pH-dependent membrane fusion, nucleocapsid disassembly, and viral genome release. These effects collectively suppress the replication and spread of enveloped viruses. The schematic also illustrates the major stages of the viral life cycle, including receptor-mediated entry, endocytosis, endosomal trafficking, genome release, viral RNA replication and transcription, protein synthesis, virion assembly, and release. Black arrows indicate the direction of viral infection and replication processes, whereas red crosses denote the stages inhibited by DNA–AuNPs.

One notable strategy is the formation of a physical barrier at the host–cell surface. As shown in Figure 5, DNA-conjugated AuNP networks assembled on the plasma membrane can act as a dynamic “nano-armor” that not only inhibits attachment of incoming virions, but also interferes with budding of newly formed viral particles [122]. Mechanistically, this surface-associated network appears to perturb membrane organization and the spatial distribution of viral components required for efficient assembly and scission, thereby reducing the release of infectious progeny. This example shows that AuNPs can modulate the virus–host interface not only through molecular recognition, but also through mesoscale control of membrane architecture.

Late-stage inhibition may also arise indirectly from AuNP-induced perturbation of intracellular trafficking and organelle dynamics. Cationic AuNPs have been reported to accumulate in lysosomes, induce lysosomal alkalinization, disrupt actin organization, and alter organelle behavior in ways that impair replication of enveloped RNA viruses [123]. Because viral assembly, maturation, and egress often depend on coordinated vesicular trafficking and membrane remodeling, such changes may secondarily compromise late stages of the viral life cycle. However, these effects should be interpreted with caution: disruption of organelle function does not automatically equate to selective antiviral activity, and excessive perturbation of host–cell physiology may also contribute to toxicity.

Additional evidence for late-stage interference comes from self-assembled plasmonic gold/layered double hydroxide composites, which significantly reduced the release of hepatitis B viral and subviral particles from infected cells [124]. In this context, the antiviral effect appears to involve intracellular retention or sequestration of viral material, thereby limiting extracellular dissemination. Although the precise mechanism may differ from that of plasma-membrane-associated nano-armor systems, both examples support the broader concept that AuNP-based materials can suppress viral propagation by obstructing the cellular pathways and membrane processes required for efficient virion release.

Overall, current evidence suggests that AuNP-mediated inhibition of viral assembly and egress can occur through at least three nonexclusive routes: alteration of host–cell membrane architecture, disruption of intracellular trafficking and organelle dynamics, and intracellular retention of viral material. Compared with entry blockade, these late-stage mechanisms are generally less sharply defined but may offer important opportunities to suppress viral spread after infection has already been established. Future progress will require more precise dissection of how specific interfacial designs influence the cellular machinery that governs virion maturation and release.

### 4.4. Photothermal Inactivation: A Physical Virucidal Modality

Photothermal inactivation represents a mechanistically distinct antiviral strategy in which AuNPs act as light-responsive energy transducers rather than as conventional ligand-based inhibitors. Owing to localized surface plasmon resonance, AuNPs can convert incident light into localized heat. This effect is particularly useful for anisotropic AuNPs, such as gold nanorods, nanostars, and nanocages, because their plasmon bands can be tuned into the near-infrared window and their high-curvature regions can enhance local electromagnetic fields and photothermal conversion efficiency [125,126,127]. In contrast, conventional spherical AuNPs generally absorb mainly in the visible region and are less efficient for NIR-triggered photothermal applications unless assembled into coupled structures or incorporated into hybrid systems [128]. Therefore, both spherical and anisotropic AuNPs can be relevant, but anisotropic structures usually provide greater advantages for photothermal antiviral design.

**Figure 6:**
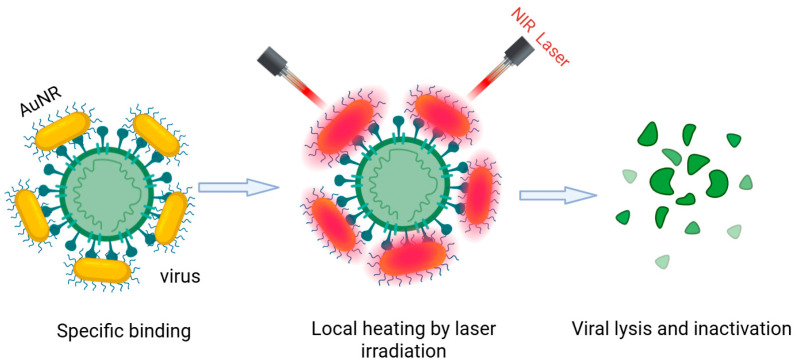
Schematic illustration of the photothermal antiviral mechanism of gold nanorods (AuNRs). AuNRs specifically bind to viral surface components and, upon near-infrared (NIR) laser irradiation, generate localized heat through the photothermal effect. The resulting thermal damage disrupts viral structural integrity, leading to structural damage and loss of infectivity.

A key advantage of photothermal antiviral systems is that their activity can be externally triggered by light. This feature distinguishes them from constitutively active viral-entry inhibitors or intracellular delivery platforms and provides spatiotemporal control over treatment location and timing. Antibody-conjugated AuNPs with spherical, star-like, and popcorn-like morphologies have been reported to inhibit human cytomegalovirus infection and photothermally damage infected cells upon irradiation [117]. Similarly, aptamer- and RVG-functionalized gold nanorods have been used to target rabies virus-infected cells in the mouse brain, where NIR-triggered photothermal treatment reduced viral burden after selective accumulation at infected sites [116]. These studies illustrate how molecular targeting and plasmonic conversion can be combined to improve treatment precision (Figure 6).

Photothermal AuNP systems may also be relevant outside the context of direct infected-cell ablation. In-solution and ex vivo applications suggest that plasmonic heating can damage viral structures more directly, provided that light delivery and nanoparticle–virus contact are adequately controlled. For example, gold and iron oxide nanoparticle assemblies constructed on turnip yellow mosaic virus have been used as model systems for in-solution photothermal experiments, demonstrating the broader feasibility of plasmonic pathogen targeting in fluid-phase settings [129]. More recently, multifunctional intranasal AuNP-based formulations have combined viral inhibition, photothermal responsiveness, and immune modulation within a single platform [130].

Despite these advantages, photothermal antiviral platforms face important translational constraints. Their efficacy depends on interdependent factors, including nanoparticle optical properties, tissue accessibility, light penetration depth, irradiation parameters, thermal confinement, and target selectivity. Excessive heating may damage healthy tissue, whereas insufficient heating may fail to inactivate virions or infected cells. Anatomical accessibility is also critical: photothermal strategies are more readily envisioned for surface-accessible tissues, localized infections, or intranasal applications than for diffuse deep-tissue viral diseases. Although anisotropic structures can partially mitigate these limitations through enhanced NIR absorption, the fundamental challenge of light delivery applies to all plasmonic nanostructures.

Overall, photothermal antiviral AuNPs demonstrate that gold nanostructures can provide therapeutic functions beyond molecular recognition and cargo delivery. By enabling externally controlled physical inactivation, they expand the mechanistic repertoire of antiviral nanomedicine. Nevertheless, their biological applicability will depend on whether their advantages in spatial precision and triggerability can outweigh limitations related to light delivery, thermal control, biodistribution, and in vivo safety. These translational hurdles are also reflected in the broader photothermal therapy field, where clinical progress remains limited despite encouraging preclinical results [131].

### 4.5. Enhancement of Prophylactic and Therapeutic Immunity

Beyond direct inhibition of viral infection, AuNPs are increasingly being developed as platforms for vaccine delivery and immune modulation. Their high surface-area-to-volume ratio, controllable nanoscale dimensions, and versatile surface chemistry allow antigens, adjuvants, and nucleic acids to be co-displayed or co-delivered in defined spatial arrangements, which can improve antigen presentation, lymphatic trafficking, and downstream immune activation. In this context, AuNPs function as tunable immune interfaces whose design can influence both the magnitude and quality of antiviral responses.

One major application is the use of AuNPs as antigen-display scaffolds. Conjugation of the conserved influenza M2e peptide to AuNPs has been shown to enhance immunogenicity relative to free peptide and to support protective responses against multiple influenza A subtypes [132,133]. The addition of soluble CpG further strengthens these effects and has been associated with durable protection in animal models [134]. AuNPs can also support more complex co-presentation strategies. For example, dual-linker AuNPs displaying recombinant hemagglutinin trimers together with flagellin combine antigen presentation with innate immune stimulation, thereby enhancing both humoral and cellular immune responses [135]. Similar design principles have also been extended to peptide-based SARS-CoV-2 vaccine constructs [136].

AuNPs may also contribute to adjuvant function more directly. Their nanoscale properties can affect uptake by antigen-presenting cells, intracellular processing, dendritic-cell maturation, and cytokine signaling, all of which may shape vaccine performance [137]. However, AuNPs should not be assumed to be intrinsically immunostimulatory across formulations. These effects can be further tuned through surface chemistry. For instance, tannic-acid-modified nanoparticles have shown enhanced adjuvant-like activity and improved antiviral immune responses in vaccination-related models [138]. At the same time, it is important to distinguish between AuNPs serving as structural carriers for defined immunogens and AuNPs contributing to immune potentiation through their physicochemical and interfacial properties.

Beyond prophylactic vaccination, AuNP-based systems may also be useful for therapeutic immune modulation during active viral infection. Lipid-encapsulated AuNPs have been developed to attenuate excessive inflammatory responses in SARS-CoV-2 infection, suggesting that AuNP formulations may help regulate virus-associated immunopathology in addition to supporting protective immunity [139]. AuNPs have also been explored as carriers for nucleic acid immunization. Polyethylenimine (PEI)-modified AuNPs can form stable complexes with plasmid DNA, improve intracellular delivery, and enhance transgene expression in vitro and in vivo [140]. More advanced formulations have combined AuNP-based antigen delivery with sustained-release formats such as chitosan microneedle patches to improve lymph-node targeting and prolong immune stimulation [141].

Overall, the immunological utility of AuNPs extends beyond passive antigen carriage. They can function as modular immune interfaces that integrate antigen presentation, adjuvant activity, nucleic acid delivery, and inflammatory regulation within a single platform. Their future development will depend on more systematic understanding of how particle size, ligand composition, administration route, and nano–bio interactions influence the quality, durability, and safety of antiviral immunity. In this respect, immunologically active AuNPs exemplify both the promise and the complexity of multifunctional antiviral nanomedicine.

### 4.6. Quantitative Trends in Representative Antiviral AuNP Studies

Although a pooled statistical meta-analysis is currently limited by the heterogeneity of virus models, nanoparticle formulations, dose units, and antiviral endpoints, several quantitative and semi-quantitative trends can still be extracted from representative studies. Table 4 summarizes reported particle sizes, surface ligands, virus models, antiviral readouts, and proposed mechanisms for selected antiviral AuNP systems. Overall, the available data suggest that antiviral activity is strongly influenced by multivalent ligand display, nanoparticle size and morphology, ligand-shell flexibility, and stability in biological media. However, many studies do not report ligand density, hydrodynamic diameter, binding affinity, or standardized selectivity indices, which remains a major barrier to direct cross-study comparison.

Although a pooled statistical meta-analysis is currently limited by the heterogeneity of virus models, nanoparticle formulations, dose units, and antiviral endpoints, representative studies provide several quantitative and semi-quantitative insights into antiviral AuNP design. Table 4 and Table 5 summarizes reported particle sizes, surface ligands, virus models, antiviral readouts, potency or efficacy information, cytotoxicity data when available, and proposed mechanisms for selected antiviral AuNP systems.

Overall, the available data suggest that antiviral activity is strongly influenced by multivalent ligand display, nanoparticle size and morphology, ligand-shell flexibility, and stability in biological media. For instance, entry-inhibiting AuNPs (Table 4) typically rely on dense ligand packing on smaller spherical cores to achieve low-nanomolar to picomolar inhibition, whereas intracellular and photothermal platforms (Table 5) require careful balancing of endosomal escape and shape-dependent plasmonic conversion. However, many studies do not report ligand density, hydrodynamic diameter, binding affinity, or standardized selectivity indices, which remains a major barrier to direct cross-study comparison.

## 5. Current Challenges and Future Research Directions

Functionalized AuNPs have emerged as highly adaptable antiviral nanoplatforms because they can integrate multiple therapeutic functions within a single construct. As discussed throughout this review, their antiviral performance is governed not only by the intrinsic properties of the gold core, but also by the architecture of the interfacial layer. Parameters such as particle size, morphology, ligand identity, surface density, linker flexibility, charge distribution, and colloidal stability collectively determine how AuNPs interact with viral particles, host–cell membranes, serum proteins, and immune components. This tunability underlies their diverse utility as entry inhibitors, intracellular delivery vehicles, photothermal agents, and vaccine or immune-modulating platforms. At the same time, it is precisely this multifunctional complexity that makes clinical translation challenging.

A first major barrier is the still-limited predictability of structure–activity relationships under physiologically relevant conditions. Although many studies demonstrate promising antiviral effects in vitro, biological performance often changes substantially in the presence of serum proteins, mucus, extracellular matrix components, or complex tissue environments. In particular, protein corona formation can alter nanoparticle identity after administration, thereby affecting ligand accessibility, biodistribution, immune recognition, and cellular uptake [80]. As a result, formulations that appear highly effective under simplified experimental conditions may behave differently in vivo. Future progress will therefore require more rigorous correlation of physicochemical design parameters with biological performance in relevant media, tissues, and animal models.

A second major challenge concerns safety, biodistribution, and long-term fate. Many AuNP formulations show acceptable short-term cytocompatibility, but this should not be taken as evidence of long-term safety across exposure routes, dosing schedules, or particle classes. Retention in organs, chronic inflammatory responses, off-target accumulation, and the biological consequences of repeated administration remain insufficiently resolved for many antiviral AuNP systems. These issues are particularly important for formulations intended for prophylactic use, repeated dosing, mucosal delivery, or immune modulation, where safety thresholds may be especially stringent. A more complete understanding of how core size, shape, surface chemistry, and degradability influence long-term in vivo behavior will be essential for translational development.

Third, the field is hindered by limited standardization across studies. Differences in synthesis, purification, ligand quantification, colloidal characterization, antiviral assay design, dose reporting, and endpoint selection make it difficult to compare results across laboratories or to derive robust general design rules. In many cases, nominally similar AuNP formulations are described without sufficiently detailed characterization of particle size distribution, surface composition, ligand density, or stability in biologically relevant media. This lack of harmonization complicates interpretation of antiviral efficacy and contributes to uncertainty regarding reproducibility. More standardized reporting frameworks and benchmarking practices will be needed to establish reliable structure–function–mechanism relationships.

A fourth barrier lies in manufacturing and scale-up. Antiviral AuNPs are often most effective when they incorporate multiple functional elements—such as stealth coatings, targeting ligands, therapeutic cargos, and trigger-responsive components—but each added layer increases synthetic complexity and potential batch variability. Large-scale production of multifunctional AuNPs with consistent size, surface composition, ligand coverage, and biological behavior remains a substantial technical challenge. In addition, purification, storage stability, sterilization, and quality control must all be optimized in ways compatible with regulatory expectations. Thus, translational success will depend not only on elegant proof-of-concept designs, but also on manufacturable and reproducible formulations.

Looking forward, three research directions appear particularly important. First, the field would benefit from more quantitatively designed multivalent interfaces that better match the geometry, spacing, and binding characteristics of viral surfaces. Second, greater emphasis should be placed on smart and context-responsive systems capable of activating drug release, structural reconfiguration, or photothermal effects selectively within infected tissues. Third, there is a pressing need for integrated evaluation pipelines that combine rigorous nanoparticle characterization with mechanistic virology, pharmacology, immunology, and long-term biosafety assessment. Advances in these areas would help shift antiviral AuNPs from isolated proof-of-concept studies toward more predictable and translationally relevant platforms.

Another important translational issue is the potential emergence of viral resistance to AuNP-based antiviral platforms. Multivalent AuNPs may impose a higher genetic barrier to resistance than conventional single-target small molecules, particularly when they target conserved host–virus interfaces, multiple spatially distributed viral surface features, or physical properties of virions. However, resistance should not be assumed to be impossible. Viruses may reduce susceptibility by altering glycosylation patterns, receptor usage, envelope-protein charge, surface accessibility, or virion stability. Future studies should evaluate resistance using serial viral passaging under subinhibitory nanoparticle concentrations, sequencing of escape populations, receptor-usage and glycosylation analyses, cross-resistance testing against related nanoparticle formulations, and fitness-cost assessment of putative escape variants.

In summary, functionalized AuNPs offer a uniquely versatile foundation for antiviral nanomedicine, but their future impact will depend on whether interfacial complexity can be converted into biological precision and manufacturable robustness. The most successful next-generation systems are likely to be those designed not only for strong antiviral activity in simplified models, but also for reproducibility, safety, and mechanistic clarity in clinically relevant settings. By addressing these challenges, AuNP-based antiviral platforms may progress from promising experimental constructs to credible candidates for broad-spectrum and precision antiviral intervention.

## Figures and Tables

**Table 1:** Synthetic and interface-engineering strategies for AuNPs used in antiviral nanoplatforms.

Synthetic Route	Typical Reaction Features	Main AuNP Characteristics	Advantages for Antiviral Nanoplatforms	Limitations/Design Concerns	References
**Turkevich method/citrate reduction**	HAuCl_4_ is reduced in an aqueous phase by heating with citrate, which serves as both a reducing agent and a weak stabilizer	Predominantly spherical, citrate-capped AuNPs (typically 10–100 nm) exhibiting high hydrophilicity and excellent dispersibility in aqueous media	This method is facile, cost-effective, highly reproducible, and compatible with post-synthetic ligand exchange, thereby facilitating PEGylation, nucleic acid conjugation, and peptide/glycan display	Limited control over anisotropic morphologies, broader size distributions at larger diameters, and a labile citrate layer that is prone to instability in high-salt or protein-rich media without further modification	[24,25,26,27,28,29,30]
**Brust–Schiffrin method/two-phase thiolate synthesis**	AuCl_4_^−^ is phase-transferred into an organic medium and subsequently reduced by NaBH_4_ in the presence of thiol ligands.	Ultrasmall thiolate-protected AuNPs or nanoclusters (typically 1–6 nm) possessing a dense and highly stable ligand shell	This route yields ultrasmall cores with high surface-to-volume ratios and robust Au-stabilization, making them highly suitable for compact intracellular delivery systems	Hydrophobic nature of the as-synthesized products requiring subsequent ligand exchange for biological applications, coupled with increased purification complexity due to organic solvents and phase-transfer reagents	[31,32,33,34,35]
**Seed-mediated growth**	Preformed Au seeds are enlarged in a growth solution containing an Au precursor, a mild reductant, a surfactant, and shape-directing additives (typically CTAB)	Tunable spherical or anisotropic AuNPs, such as nanorods, nanostars, and other plasmonic nanostructures	This approach offers precise control over morphology and optical properties, which is highly advantageous for developing NIR-responsive photothermal antiviral platforms	Potential cytotoxicity of residual CTAB or other surfactants, reduced reproducibility inherent to multistep syntheses, and the necessity for extensive purification and surface exchange	[36,37,38,39,40,41]
**Green synthesis/biogenic synthesis**	Biological extracts, microorganisms, enzymes, or biomolecule-rich media are utilized to reduce Au^3+^ and stabilize the resulting AuNPs	AuNPs capped with phytochemicals, proteins, polysaccharides, or metabolites, with their size and shape varying depending on the biological source	The synthesis is mild, sustainable, and highly biocompatible, wherein natural capping layers can exert synergistic antiviral, antioxidant, or anti-inflammatory therapeutic effects	Limited mechanistic control, pronounced batch-to-batch variability, poorly defined surface compositions, and significant challenges in standardization and scale-up	[42,43,44,45,46,47]
**Direct thiol-conjugation synthesis**	Thiol-containing ligands are introduced during nucleation/growth or immediately after particle formation to achieve direct Au-S covalent anchoring	AuNPs functionalized with predefined terminal groups, including PEG, amine, carboxyl, glycan, peptide, or cationic polymer shells	This strategy couples core formation with interfacial programming, thereby enhancing control over surface charge, hydrophilicity, ligand density, and functional group presentation	Strong dependence of reaction conditions on ligand structures, potential restriction of particle growth or heterogeneity caused by excessive thiol binding, and the critical need for ligand-density optimization to ensure biological efficacy	[49,50,51,52,53]
**Mixed-ligand shell strategy**	Two or more distinct ligands are co-assembled on the AuNP surface via ligand exchange, direct synthesis, or sequential conjugation	Multifunctional AuNPs integrating both stealth properties and bioconjugation-functional components	This approach balances competing requirements (e.g., antifouling behavior versus bioconjugation functionality) and enables highly modular surface engineering	The requirement for precise control over ligand ratios to ensure batch reproducibility, and the risk of reduced active-ligand accessibility due to competitive ligand adsorption	[54,55,56,57,58,59]

**Table 2:** Effects of representative surface functionalization strategies on corona formation, protein resistance, and antiviral performance of AuNPs.

Surface Functionalization Strategy	Main Corona-Regulating Feature	Effect on Protein Adsorption/Biological Identity	Relevance to Antiviral AuNPs	Main Limitations	References
PEGylation	Hydrated steric barrier	Minimizes nonspecific protein adsorption and aggregation, particularly when optimal chain lengths and high grafting densities are employed	Enhances colloidal stability, prolongs systemic circulation lifetime, and improves the steric accessibility of co-displayed antiviral ligands	Incomplete inertness, with the potential occurrence of residual protein adsorption and anti-PEG immunological responses	[60,61,62,63,64,103,104]
Zwitterionic or mixed-charge coatings	Strong hydration layer and balanced local charges	Suppresses nonspecific electrostatic interactions, thereby significantly reducing macrophage-mediated uptake	Preserves robust antiviral activity even within highly complex, protein-rich physiological environments	The stringent requirement for precise control over polymer chemistry, grafting density, and ligand ratios	[105]
Anionic surfaces, including citrate, carboxylate, sulfate, or sulfonate groups	Hydrophilic charged interface; possible receptor-mimetic binding	Enhances colloidal stability, albeit with persistent adsorption of serum proteins	Mimics HSPG-like viral entry pathways and facilitates multivalent receptor-mimetic binding	Masking of active anionic ligands by the protein corona, potentially altering or compromising viral binding affinity	[18,58]
Cationic or amine-rich surfaces	Electrostatic interaction with membranes and proteins	Typically promotes protein adsorption, cellular internalization, and macrophage recognition, with an elevated risk of cytotoxicity	Facilitates efficient intracellular delivery and robust nucleic acid complexation	Elevated nonspecific adsorption, high risk of systemic toxicity, and accelerated physiological clearance	[47,106,107]
DNA/RNA functionalization	Hydrophilic, highly charged oligonucleotide shell	Modulates protein corona composition and provides partial steric/electrostatic shielding	Enables targeted siRNA delivery, aptamer-mediated homing, and spherical nucleic acid (SNA)-like antiviral configurations	Pronounced dependence of corona behavior on oligonucleotide sequence, grafting density, hybridization state, and media composition	[65,66,67,68,69,70,71,72,73]
Protein, antibody, nanobody, or peptide ligands	Biological recognition layer	Confers high target specificity, which may nevertheless be masked by the adsorption of extraneous serum proteins	Enables precise targeting of virions or infected cells, leading to multivalent viral neutralization	Potential loss of bioactivity due to unfavorable ligand orientation, denaturation, steric crowding, or corona-mediated masking	[75,76,77,78,79,80]
Polysaccharide or glycan coatings	Hydrophilic carbohydrate-rich interface; receptor mimicry	Can modulate protein adsorption depending on charge, density, and glycan structure	Useful for glycan-mediated viral attachment blockade and receptor mimicry	Glycan ligands may be masked by serum proteins; activity depends on presentation	[48,85,86,87,88,89,90,91,92]

**Table 4:** Representative entry-inhibiting and virucidal AuNP systems and their reported antiviral trends.

AuNP Design/Surface Ligand	Size or Morphology	Virus Model	Experimental System	Reported Quantitative/Semi-Quantitative Readout	Main Design Implication	Ref.
Sulfate-ended ligand-capped AuNPs	~2 nm AuNP cores	HIV-1	In vitro infection assay	IC_50_ = **1.29 µg/mL** (0.012 µM NP) for 100% sulfated AuNPs; IC_50_ = **2.32 µg/mL** for 50% sulfated; free ligand >10 µg/mL	Dense anionic sulfate ligands target basic residues within the gp120 V3 loop, achieving multivalent blockade of viral entry	[18]
Mercaptoethane sulfonate-capped AuNPs	~4 nm AuNPs	HSV-1	In vitro	Complete blockage of plaque formation at **400 µg/mL** (42–4200 PFU); **0 PFU** recovered in end-point titration at MOI 20–70 (Vero/HSV-1 McIntyre). No exact IC_50_ reported	Sulfonated AuNPs are capable of blocking multiple early stages of viral infection	[19]
Sialic-acid-functionalized AuNPs	Size-dependent spherical AuNP series	Influenza virus	Binding and in vitro infection assays	**~40% reduction** in MDCK cell infection (14 nm SA-AuNP, MOI 25, X31)	Nanoparticle size and ligand presentation cooperatively modulate hemagglutinin-mediated multivalent binding	[20]
Polyvalent nano-lectin AuNPs	13 nm AuNPs	SARS-CoV-2 variants	Pseudovirus and live-virus-related assays	Kd **< 1 nM** (glycan-QD binding); Pseudovirus EC_50_ **low nM**; Authentic SARS-CoV-2 EC_50_ **< 10 pM** (B.1) and **<10 nM** (BA.1 Omicron)	Multivalent lectin display significantly enhances otherwise weak monomeric lectin–glycan interactions	[108]
Glycomimetic AuNPs	∼5 nm and ∼13 nm AuNPs	Ebola pseudovirus (EBOVpp) glycan-recognition model	Biophysical and viral-entry assays	G5- and G13 psDiMan-50% and 100% potently blocked cell surface DC-SIGN-promoted cell entry of EBOV_pp_, with EC_50_ values being determined as 0.43 ± 0.17, 0.06 ± 0.03, 0.49 ± 0.13, and 0.18 ± 0.04 nM	Ligand density, spatial spacing, and nanoscale architecture cooperatively regulate antiviral potency	[109]
Glycan-AuNP probes	~5 nm core	Ebola pseudoviruses	Binding and blocking assays	GNP-glycans can potently block DC-SIGN-mediated EBOV-GP driven viral infections of host cells with IC_50_ values down to 95 ± 17 pM	Multivalent glycan display serves as a robust design principle for blocking viral entry	[110]
Peptide fusion inhibitor AuNPs	2 nm AuNPs	HIV-1	In vitro fusion assay	IC_50_ = **10 nM** (SDC-1721-NP, 12:1 loading); free SDC-1721 **inactive**; TAK-779 control IC_50_ = **10 nM** (PBMC/HIV-1 JR-CSF CCR5, day 7 p24 ELISA)	Multivalent peptide presentation increases local inhibitor density	[83]
Poly(styrene sulfonate)-coated AuNPs	~3 nm core	HSV-2, RSV	In vitro infectivity and virucidal assays	IC_50_ = **89.91 ng/mL** (PSS-AuNP, HSV-2 plaque assay); **>99% (>2log_10_)** PFU reduction in serial dilution confirming virucidal mechanism; free L-PSS IC_50_ = 27.90 ng/mL with only 66% reduction (virustatic)	Nanoparticle-templated presentation can successfully transition soluble polyanions into highly active virucidal interfaces	[113]
Sulfonic mixed-charge modified AuNPs	∼6 nm and ~16 nm AuNPs	Lentiviral vectors or AAV5 model systems	In vitro high-protein assay	IC_50_ = **12.82 µg/mL** (6 nm MC_AuNP MDS:TMA = 8:2) vs. **21.64 µg/mL** (single-sulfonic MDS_AuNP) in protein-free condition; MC_AuNP retained >80% inhibition in 10 mg/mL BSA, while MDS_AuNP lost all activity at 1 mg/mL BSA (293T/lentivirus-GFP)	A mixed-charge surface design significantly improves nanoparticle robustness against protein-corona-mediated masking	[105]
Polyaniline AuNPs	Porous AuNPs	Influenza A virus	Infection and hemagglutinin-function assays	Cell viability increased from **33.9%** (untreated) to **74–76%** (0.2 mg/mL PoGNP, 60 min) across H1N1/H3N2; intracellular viral RNA significantly reduced by RT-qPCR; non-porous sGNP showed no antiviral effect. No IC_50_ reported	Porous gold surfaces are capable of perturbing the function of viral envelope glycoproteins	[114]

**Table 5:** Representative intracellular delivery, photothermal, and vaccine-related AuNP systems and their reported antiviral trends.

AuNP Design	Size or Morphology	Virus/Model	Evidence Level	Reported Quantitative/Semi-Quantitative Readout	Main Design Implication	Ref.
siRNA-functionalized AuNPs	~13 nm AuNPs,	Dengue virus	In vitro	~2–15 fold DENV-2 FFU reduction (varies by formulation); LDH cytotoxicity ≤6.5% for all formulations; AuNP size ~15–40 nm. No IC_50_/EC_50_ reported.	AuNPs protect fragile siRNA payloads from enzymatic degradation and enhance their intracellular delivery	[71]
Ultrasmall AuNP–siRNA conjugates	~2 nm AuNPs	HSV/NF-κB signaling	In vitro	Complete HSV-2 inhibition at IC_100_ = 2 pmol/mL (Au-siRNA-18.1) and 4 pmol/mL (Au-siRNA-29.2); siRNA loading 6–10 molecules per 2 nm AuNP; NF-κB gene silencing shown by fold change	Ultrasmall AuNPs enable the co-delivery of therapeutic agents for combined antiviral and host-modulatory RNAi	[72]
Tenofovir-tethered AuNPs	~25 nm AuNPs	HIV-related model	In vivo	IC_50_ = 0.016–0.037 µg/mL (AuNP-TNF, PBMC/TZM-bl, R5/X4 HIV-1); ~15-fold more potent than free TNF; RT-ase IC_50_ = 0.067 µg/mL; TI max = 22,897 (PBMC, R5); drug conjugation ~16 wt%; cellular uptake 78.9% at 120 min.	Covalent conjugation of drugs to AuNPs can prolong drug residence time and optimize therapeutic delivery	[93]
Aptamer/RVG-functionalized AuNRs	Gold nanorods	Rabies virus	In vivo mouse brain model	~100-fold intracellular virus titer decrease after 5 min NIR; 65.2% apoptosis of infected cells; in vivo survival 60% (PTT) vs. 0% (untreated); photothermal temperature 51.3 °C at 2.5 mM	Covalent conjugation of drugs to AuNPs can prolong drug residence time and optimize therapeutic delivery	[116]
Bioconjugated photothermal AuNPs	Spherical, star-like, and popcorn-like AuNPs	Human cytomegalovirus	In vitro infected-cell model	gB-functionalized 8 nm GNPs achieved **≥10,000-fold reduction in viral yield** upon 532 nm laser irradiation (5 min, reaching ~50 °C), with no cytotoxicity observed in the absence of laser treatment.	Nanoparticle morphology and ligand-mediated targeting cooperatively dictate photothermal antiviral efficacy	[117]
M2e peptide-conjugated AuNPs	~12 nm AuNPs	Influenza A virus	Animal immunization models	Consensus M2e peptides displayed on 12 nm AuNPs with sCpG adjuvant conferred 100% survival against H1N1 and H5N1 lethal challenge (3 × LD_50_) and 92% survival against H3N2. Protection was sustained at 100% up to 8 months after a single vaccination, with minimal body weight loss (~4%). In aged mice, M2e-specific antibody responses and functional memory B cells persisted beyond 15 months, and passive serum transfer conferred protection to naive recipients.	Nanoparticle-mediated antigen display significantly enhances immunogenicity and elicits robust protective immunity	[132,133,134]

## Data Availability

No new data were created or analyzed in this study. Data sharing is not applicable to this article.

## References

[1] Aw D.Z.H., Zhang D.X., Vignuzzi M. (2025). Strategies and efforts in circumventing the emergence of antiviral resistance against conventional antivirals. npj Antimicrob. Resist..

[2] Gupta R.K., Hill A., Sawyer A.W., Cozzi-Lepri A., von Wyl V., Yerly S., Lima V.D., Günthard H.F., Gilks C., Pillay D. (2009). Virological monitoring and resistance to first-line highly active antiretroviral therapy in adults infected with HIV-1 treated under WHO guidelines: A systematic review and meta-analysis. Lancet Infect. Dis..

[3] Du S., Hu X., Li P., Xu S., Kim M., Liu X., Zhan P. (2026). Antiviral drug discovery and development: Challenges and future directions. Signal Transduct. Target. Ther..

[4] Xin G.-L., Zhang C., Ni J.-L., Li Y.-K., Sun Y., He X.-X. (2025). Nanomaterial Applications in Prevention and Treatment Strategies of Virus: A Review. Bioconjug. Chem..

[5] Huang Y., Guo X., Wu Y., Chen X., Feng L., Xie N., Shen G. (2024). Nanotechnology’s frontier in combatting infectious and inflammatory diseases: Prevention and treatment. Signal Transduct. Target. Ther..

[6] Jeevanandam J., Krishnan S., Hii Y.S., Pan S., Chan Y.S., Acquah C., Danquah M.K., Rodrigues J. (2022). Synthesis approach-dependent antiviral properties of silver nanoparticles and nanocomposites. J. Nanostruct. Chem..

[7] Nakano R., Nakano A., Sasahara T., Suzuki Y., Nojima Y., Yano H. (2025). Antiviral effects of copper and copper alloy and the underlying mechanisms in severe acute respiratory syndrome coronavirus 2. J. Hazard. Mater. Adv..

[8] Aich P., Chung W.P., Wang W.J., Takahashi H., Hsieh M.S., Lin Y.C., Kwon O.S., Li W.P., Huang W.L. (2026). Biomimetic Copper Nanoparticles Coated with ACE2-Overexpressing Membranes for Selective SARS-CoV-2 Neutralization and Disinfection. Adv. Healthc. Mater..

[9] Gutiérrez Rodelo C., Salinas R.A., Armenta Jaime E., Armenta S., Galdámez-Martínez A., Castillo-Blum S.E., Astudillo-de la Vega H., Nirmala Grace A., Aguilar-Salinas C.A., Gutiérrez Rodelo J. (2022). Zinc associated nanomaterials and their intervention in emerging respiratory viruses: Journey to the field of biomedicine and biomaterials. Coord. Chem. Rev..

[10] Zhou Y., Tong T., Jiang X., Fang L., Wu Y., Liang J., Xiao S. (2020). GSH-ZnS Nanoparticles Exhibit High-Efficiency and Broad-Spectrum Antiviral Activities via Multistep Inhibition Mechanisms. ACS Appl. Bio Mater..

[11] El-Zahed M.M., Kandel S.A., Khalifa M.E. (2026). Antiviral activity of green synthesized selenium nanoparticles alone and in combination with chitosan against SARS-CoV-2. Nanoscale Res. Lett..

[12] Su J., Lai J., Li J., Li C., Liu X., Wang C., Zhu B., Li Y. (2023). Selenium Nanoparticles Control H1N1 Virus by Inhibiting Inflammatory Response and Cell Apoptosis. Molecules.

[13] Zhou Y., Jiang X., Tong T., Fang L., Wu Y., Liang J., Xiao S. (2020). High antiviral activity of mercaptoethane sulfonate functionalized Te/BSA nanostars against arterivirus and coronavirus. RSC Adv..

[14] Tong T., Deng S., Zhang X., Fang L., Liang J., Xiao S. (2022). Inhibitory effect and mechanism of gelatin stabilized ferrous sulfide nanoparticles on porcine reproductive and respiratory syndrome virus. J. Nanobiotechnol..

[15] Osminkina L.A., Agafilushkina S.N., Kropotkina E.A., Saushkin N.Y., Bozhev I.V., Abramchuk S.S., Samsonova J.V., Gambaryan A.S. (2022). Antiviral adsorption activity of porous silicon nanoparticles against different pathogenic human viruses. Bioact. Mater..

[16] Xu C., Zhang Y., Zhang C., Gao X. (2026). Gold nanoclusters—A promising atomically precise atomic aggregation-based drug and its biomedical applications. Nano Today.

[17] Teimouri H., Taheri S., Saidabad F.E., Nakazato G., Maghsoud Y., Babaei A. (2025). New insights into gold nanoparticles in virology: A review of their applications in the prevention, detection, and treatment of viral infections. Biomed. Pharmacother..

[18] Di Gianvincenzo P., Marradi M., Martínez-Ávila O.M., Bedoya L.M., Alcamí J., Penadés S. (2010). Gold nanoparticles capped with sulfate-ended ligands as anti-HIV agents. Bioorg. Med. Chem. Lett..

[19] Baram-Pinto D., Shukla S., Gedanken A., Sarid R. (2010). Inhibition of HSV-1 Attachment, Entry, and Cell-to-Cell Spread by Functionalized Multivalent Gold Nanoparticles. Small.

[20] Papp I., Sieben C., Ludwig K., Roskamp M., Böttcher C., Schlecht S., Herrmann A., Haag R. (2010). Inhibition of Influenza Virus Infection by Multivalent Sialic-Acid-Functionalized Gold Nanoparticles. Small.

[21] Forsyth C.M., Chan R.R., Fink T.D., Kang J., Cohen J.D., Petrosko S.H., Mirkin C.A. (2026). Spherical Nucleic Acids: Turning Synthetic Advances and Fundamental Discovery into Translational Breakthroughs in Chemistry, Materials Development, Biology, and Medicine. Acc. Chem. Res..

[22] Sati A., Mali S.N., Samdani N., Annadurai S., Dongre R., Satpute N., Ranade T.N., Pratap A.P. (2025). From Past to Present: Gold Nanoparticles (AuNPs) in Daily Life—Synthesis Mechanisms, Influencing Factors, Characterization, Toxicity, and Emerging Applications in Biomedicine, Nanoelectronics, and Materials Science. ACS Omega.

[23] Patil T., Gambhir R., Vibhute A., Tiwari A.P. (2023). Gold Nanoparticles: Synthesis Methods, Functionalization and Biological Applications. J. Clust. Sci..

[24] Turkevich J., Stevenson P.C., Hillier J. (1951). A study of the nucleation and growth processes in the synthesis of colloidal gold. Discuss. Faraday Soc..

[25] Frens G. (1973). Controlled Nucleation for the Regulation of the Particle Size in Monodisperse Gold Suspensions. Nat. Phys. Sci..

[26] Kimling J., Maier M., Okenve B., Kotaidis V., Ballot H., Plech A. (2006). Turkevich method for gold nanoparticle synthesis revisited. Phys. Chem. B.

[27] Tyagi H., Kushwaha A., Kumar A., Aslam M. (2016). A Facile pH Controlled Citrate-Based Reduction Method for Gold Nanoparticle Synthesis at Room Temperature. Nanoscale Res. Lett..

[28] Dong J., Carpinone P.L., Pyrgiotakis G., Demokritou P., Moudgil B.M. (2020). Synthesis of Precision Gold Nanoparticles Using Turkevich Method. Kona Powder Part. J..

[29] Salloum S., Rüther J., Celik Z., Janiak C. (2025). Comparative analysis of synthesis techniques for citrate-capped gold nanoparticles: Insights into optimized wet-chemical approaches for controlled morphology and stability. Nanoscale.

[30] Sibug-Torres S.M., Wallace G.Q., Jones T., Graham D., Baumberg J.J. (2026). The role of Au–Cl adlayers in the Turkevich synthesis of gold nanoparticles. Chem. Commun..

[31] Jin R. (2010). Quantum sized, thiolate-protected gold nanoclusters. Nanoscale.

[32] Perala S.R.K., Kumar S. (2013). On the Mechanism of Metal Nanoparticle Synthesis in the Brust–Schiffrin Method. Langmuir.

[33] Li Y., Zaluzhna O., Xu B., Gao Y., Modest J.M., Tong Y.J. (2011). Mechanistic Insights into the Brust−Schiffrin Two-Phase Synthesis of Organo-chalcogenate-Protected Metal Nanoparticles. J. Am. Chem. Soc..

[34] Zhu L., Zhang C., Guo C., Wang X., Sun P., Zhou D., Chen W., Xue G. (2013). New Insight into Intermediate Precursors of Brust–Schiffrin Gold Nanoparticles Synthesis. J. Phys. Chem. C.

[35] Booth S.G., Uehara A., Chang S.Y., La Fontaine C., Fujii T., Okamoto Y., Imai T., Schroeder S.L.M., Dryfe R.A.W. (2017). The significance of bromide in the Brust–Schiffrin synthesis of thiol protected gold nanoparticles. Chem. Sci..

[36] Zhao P., Li N., Astruc D. (2013). State of the art in gold nanoparticle synthesis. Coord. Chem. Rev..

[37] Xia Y., Gilroy K.D., Peng H.-C., Xia X. (2017). Seed-Mediated Growth of Colloidal Metal Nanocrystals. Angew. Chem. Int. Ed..

[38] Niu W., Zhang L., Xu G. (2013). Seed-mediated growth of noble metal nanocrystals: Crystal growth and shape control. Nanoscale.

[39] Zürbes K.R., Mani E., Bandyopadhyay S. (2025). Synthesis of anisotropic gold nanoparticles in binary surfactant mixtures: A review on mechanisms of particle formation. RSC Adv..

[40] Wei M.-Z., Deng T.-S., Zhang Q., Cheng Z., Li S. (2021). Seed-Mediated Synthesis of Gold Nanorods at Low Concentrations of CTAB. ACS Omega.

[41] Wang K.-P., Liu J., Chen X., Wu G.-L., Zhang E.-J., Deng T.-S. (2026). Two-step seed-mediated growth for the reproducible synthesis of high quality gold nanorods. J. Chem. Sci..

[42] Belew A.A., Gebre S.H., Assege M.A., Meshesha D.S., Ayana M.T. (2025). Biosynthesis of gold nanoparticle: Current applications, challenges, and future prospects. Results Chem..

[43] Jadoun S., Arif R., Jangid N.K., Meena R.K. (2021). Green synthesis of nanoparticles using plant extracts: A review. Environ. Chem. Lett..

[44] Mehra V., Kumar S., Tamang A.M., Chandraker S.K. (2024). Green Synthesis of Gold Nanoparticles (AuNPs) by Using Plant Extract and Their Biological Application: A Review. BioNanoScience.

[45] Santhosh P.B., Genova J., Chamati H. (2022). Green Synthesis of Gold Nanoparticles: An Eco-Friendly Approach. Chemistry.

[46] Jannathul Firdhouse M., Lalitha P. (2022). Biogenic green synthesis of gold nanoparticles and their applications—A review of promising properties. Inorg. Chem. Commun..

[47] Meléndez-Villanueva M.A., Morán-Santibañez K., Martínez-Sanmiguel J.J., Rangel-López R., Garza-Navarro M.A., Rodríguez-Padilla C., Zarate-Triviño D.G., Trejo-Ávila L.M. (2019). Virucidal Activity of Gold Nanoparticles Synthesized by Green Chemistry Using Garlic Extract. Viruses.

[48] Halder A., Das S., Ojha D., Chattopadhyay D., Mukherjee A. (2018). Highly monodispersed gold nanoparticles synthesis and inhibition of herpes simplex virus infections. Mat. Sci. Eng. C-Mater..

[49] Sugie A., Somete T., Matsubara M., Kanie K., Muramatsu A., Mori A. (2009). Generation of Gold Nanoparticles via Direct Thiol-Capping with THP-Protected Thiols without Deprotection. Synlett.

[50] Lohse S.E., Dahl J.A., Hutchison J.E. (2010). Direct Synthesis of Large Water-Soluble Functionalized Gold Nanoparticles Using Bunte Salts as Ligand Precursors. Langmuir.

[51] Xu X., Liu Y., Yang Y., Wu J., Cao M., Sun L. (2022). One-pot synthesis of functional peptide-modified gold nanoparticles for gene delivery. Colloids Surf. A.

[52] Ncobeni N., de la Torre B.G., Albericio F., Kruger H.G., Parboosing R. (2022). Active targeting of CD4+ T lymphocytes by PEI-capped, peptide-functionalized gold nanoparticles. Nanotechnology.

[53] Adokoh C.K., Keter F.K., Kinfe H.H., Tshikhudo R., Darkwa J. (2020). Development and characterization of functionalized glyco thiolate capped gold nanoparticles for biological applications. RSC Med. Chem..

[54] Oh E., Susumu K., Jain V., Kim M., Huston A. (2012). One-pot aqueous phase growth of biocompatible 15–130 nm gold nanoparticles stabilized with bidentate PEG. J. Colloid Interface Sci..

[55] Temur N., Dadi S., Dogan A.N., Nisari M., Avan I., Ocsoy I. (2025). Benefiting from Both Ethanol Oxidation and Bidentate Thiol Groups of DHLA Ligands under Photoirradiation for Synthesis of Au Nanoparticles with Their Catalytic and Peroxidase Like Activity. ACS Omega.

[56] Susumu K., Stewart M.H., Meares A., Medintz I.L., Oh E. (2025). Thioctic Acid-Based Compact Hydrophilic Ligands for Biocompatible Quantum Dots and Gold Nanoparticles: Facile Synthesis and Improved Utility. Chem. Mater..

[57] Borsley S., Edwards W., Mati I.K., Poss G., Diez-Castellnou M., Marro N., Kay E.R. (2023). A General One-Step Synthesis of Alkanethiyl-Stabilized Gold Nanoparticles with Control over Core Size and Monolayer Functionality. Chem. Mater..

[58] Cagno V., Andreozzi P., D’Alicarnasso M., Jacob Silva P., Mueller M., Galloux M., Le Goffic R., Jones S.T., Vallino M., Hodek J. (2018). Broad-spectrum non-toxic antiviral nanoparticles with a virucidal inhibition mechanism. Nat. Mater..

[59] Raza S., Mente P., Kamiński B., Bończak B., Maleki-Ghaleh H., Vignesh V., Paczesny J. (2025). Engineering hydrophobic and electrostatic interactions for selective inactivation of bacteriophages by mixed-ligand nanoparticles. Nanoscale.

[60] Nguyenova H.Y., Hubalek Kalbacova M., Dendisova M., Sikorova M., Jarolimkova J., Kolska Z., Ulrychova L., Weber J., Reznickova A. (2024). Stability and biological response of PEGylated gold nanoparticles. Heliyon.

[61] Suk J.S., Xu Q., Kim N., Hanes J., Ensign L.M. (2016). PEGylation as a strategy for improving nanoparticle-based drug and gene delivery. Adv. Drug Deliv. Rev..

[62] Maus L., Dick O., Bading H., Spatz J.P., Fiammengo R. (2010). Conjugation of Peptides to the Passivation Shell of Gold Nanoparticles for Targeting of Cell-Surface Receptors. ACS Nano.

[63] Fujiura K., Naito M., Tanaka Y., Tanaka M., Nakanishi Y., Ejima H., Negishi L., Kujirai T., Kurumizaka H., Ohta S. (2024). Development of stealth nanoparticles coated with poly(2-methoxyethyl vinyl ether) as an alternative to poly(ethylene glycol). J. Appl. Polym. Sci..

[64] Kozma G.T., Shimizu T., Ishida T., Szebeni J. (2020). Anti-PEG antibodies: Properties, formation, testing and role in adverse immune reactions to PEGylated nano-biopharmaceuticals. Adv. Drug Deliv. Rev..

[65] Liu B., Liu J. (2017). Methods for preparing DNA-functionalized gold nanoparticles, a key reagent of bioanalytical chemistry. Anal. Methods.

[66] Gorbunova E.A., Epanchintseva A.V., Pyshnyi D.V., Pyshnaya I.A. (2023). Noncovalent Adsorption of Single-Stranded and Double-Stranded DNA on the Surface of Gold Nanoparticles. Appl. Sci..

[67] Carnerero J.M., Jimenez-Ruiz A., Castillo P.M., Prado-Gotor R. (2017). Covalent and Non-Covalent DNA–Gold-Nanoparticle Interactions: New Avenues of Research. ChemPhysChem.

[68] Ding Y., Jiang Z., Saha K., Kim C.S., Kim S.T., Landis R.F., Rotello V.M. (2014). Gold Nanoparticles for Nucleic Acid Delivery. Mol. Ther..

[69] Sun M., Wu Z., Zhang J., Chen M., Lu Y., Yang C., Song Y. (2022). Spherical neutralizing aptamer suppresses SARS-CoV-2 Omicron escape. Nano Today.

[70] Xia C., Cheng H., Hou X., Zhang Y., Zhou X., Yan Q., Cao S. (2024). Spherical nucleic acids for biomedical applications. Adv. Sens. Energy Mater..

[71] Paul A.M., Shi Y., Acharya D., Douglas J.R., Cooley A., Anderson J.F., Huang F., Bai F. (2014). Delivery of antiviral small interfering RNA with gold nanoparticles inhibits dengue virus infection in vitro. J. Gen. Virol..

[72] Wolff N., Kollenda S., Klein K., Loza K., Heggen M., Brochhagen L., Witzke O., Krawczyk A., Hilger I., Epple M. (2022). Silencing of proinflammatory NF-κB and inhibition of herpes simplex virus (HSV) replication by ultrasmall gold nanoparticles (2 nm) conjugated with small-interfering RNA. Nanoscale Adv..

[73] Giljohann D.A., Seferos D.S., Prigodich A.E., Patel P.C., Mirkin C.A. (2009). Gene Regulation with Polyvalent siRNA−Nanoparticle Conjugates. J. Am. Chem. Soc..

[74] Shiang Y.-C., Ou C.-M., Chen S.-J., Ou T.-Y., Lin H.-J., Huang C.-C., Chang H.-T. (2013). Highly efficient inhibition of human immunodeficiency virus type 1 reverse transcriptase by aptamers functionalized gold nanoparticles. Nanoscale.

[75] Jazayeri M.H., Amani H., Pourfatollah A.A., Pazoki-Toroudi H., Sedighimoghaddam B. (2016). Various methods of gold nanoparticles (GNPs) conjugation to antibodies. Sens. Bio-Sens. Res..

[76] Ruiz G., Tripathi K., Okyem S., Driskell J.D. (2019). pH Impacts the Orientation of Antibody Adsorbed onto Gold Nanoparticles. Bioconjug. Chem..

[77] Sotnikov D.V., Berlina A.N., Ivanov V.S., Zherdev A.V., Dzantiev B.B. (2019). Adsorption of proteins on gold nanoparticles: One or more layers?. Colloids Surf. B.

[78] Song T., Cooper L., Galván Achi J., Wang X., Dwivedy A., Rong L., Wang X. (2024). Polyvalent Nanobody Structure Designed for Boosting SARS-CoV-2 Inhibition. J. Am. Chem. Soc..

[79] Dominguez-Medina S., Blankenburg J., Olson J., Landes C.F., Link S. (2013). Adsorption of a Protein Monolayer via Hydrophobic Interactions Prevents Nanoparticle Aggregation under Harsh Environmental Conditions. ACS Sustain. Chem. Eng..

[80] Halder K., Dasgupta S. (2025). Temperature dependent human serum albumin Corona formation: A case study on gold nanorods and nanospheres. Int. J. Biol. Macromol..

[81] Shirazi A.N., Vadlapatla R., Koomer A., Nguyen A., Khoury V., Parang K. (2025). Peptide-Based Inorganic Nanoparticles as Efficient Intracellular Delivery Systems. Pharmaceutics.

[82] Liu X., Zhang Q., Knoll W., Liedberg B., Wang Y. (2020). Rational Design of Functional Peptide–Gold Hybrid Nanomaterials for Molecular Interactions. Adv. Mater..

[83] Bowman M.-C., Ballard T.E., Ackerson C.J., Feldheim D.L., Margolis D.M., Melander C. (2008). Inhibition of HIV Fusion with Multivalent Gold Nanoparticles. J. Am. Chem. Soc..

[84] Kotelnikov I., Mondo G.B., da Silva Ribeiro C.A., Sosa M.M.R., Alves W.A., Batista B.L., Pop-Georgievski O., Proks V., Giacomelli C., Giacomelli F.C. (2026). Building a Simple Platform for Tailoring Peptide Surface Chemistry to Enhance Cellular Uptake of Polymer-Coated Gold Nanoparticles. ACS Omega.

[85] Mathez G., Cagno V. (2021). Viruses Like Sugars: How to Assess Glycan Involvement in Viral Attachment. Microorganisms.

[86] García I., Marradi M., Penadés S. (2010). Glyconanoparticles: Multifunctional Nanomaterials for Biomedical Applications. Nanomedicine.

[87] Sapsford K.E., Algar W.R., Berti L., Gemmill K.B., Casey B.J., Oh E., Stewart M.H., Medintz I.L. (2013). Functionalizing Nanoparticles with Biological Molecules: Developing Chemistries that Facilitate Nanotechnology. Chem. Rev..

[88] Wang X., Matei E., Deng L., Ramström O., Gronenborn A.M., Yan M. (2011). Multivalent glyconanoparticles with enhanced affinity to the anti-viral lectin Cyanovirin-N. Chem. Commun..

[89] Chen X., Zhao X., Wang G. (2020). Review on marine carbohydrate-based gold nanoparticles represented by alginate and chitosan for biomedical application. Carbohydr. Polym..

[90] Sun X.-L. (2021). The role of cell surface sialic acids for SARS-CoV-2 infection. Glycobiology.

[91] Basaran R., Budhadev D., Dimitriou E., Wootton H.S., Miller G.J., Kempf A., Nehlmeier I., Pöhlmann S., Guo Y., Zhou D. (2025). Polyvalent Mannuronic Acid-Coated Gold Nanoparticles for Probing Multivalent Lectin–Glycan Interaction and Blocking Virus Infection. Viruses.

[92] Tong T., Zhang X., Lei Y., Li L., Xiao S., Liang J. (2026). Glycyrrhizic Acid-Modified Gold Nanoparticles Show Inhibitory Activity Against PRRSV and SARS-CoV-2 Pseudovirus In Vitro. Viruses.

[93] Fotooh Abadi L., Kumar P., Paknikar K., Gajbhiye V., Kulkarni S. (2023). Tenofovir-tethered gold nanoparticles as a novel multifunctional long-acting anti-HIV therapy to overcome deficient drug delivery-: An in vivo proof of concept. J. Nanobiotechnol..

[94] Georgeous J., AlSawaftah N., Abuwatfa W.H., Husseini G.A. (2024). Review of Gold Nanoparticles: Synthesis, Properties, Shapes, Cellular Uptake, Targeting, Release Mechanisms and Applications in Drug Delivery and Therapy. Pharmaceutics.

[95] Song L., Ho V.H.B., Chen C., Yang Z., Liu D., Chen R., Zhou D. (2013). Efficient, pH-Triggered Drug Delivery Using a pH-Responsive DNA-Conjugated Gold Nanoparticle. Adv. Healthc. Mater..

[96] Park J., Han H., Ahn J.K. (2024). Development of Targeted Drug Delivery System for the Treatment of SARS-CoV-2 Using Aptamer-Conjugated Gold Nanoparticles. Pharmaceutics.

[97] Amina S.J., Guo B. (2020). A Review on the Synthesis and Functionalization of Gold Nanoparticles as a Drug Delivery Vehicle. Int. J. Nanomed..

[98] Yoon B.K., Jeon W.-Y., Sut T.N., Cho N.-J., Jackman J.A. (2021). Stopping Membrane-Enveloped Viruses with Nanotechnology Strategies: Toward Antiviral Drug Development and Pandemic Preparedness. ACS Nano.

[99] Kenry (2024). Microfluidic-assisted formulation of cell membrane-camouflaged anisotropic nanostructures. Nanoscale.

[100] Zhang Y., Cai K., Li C., Guo Q., Chen Q., He X., Liu L., Zhang Y., Lu Y., Chen X. (2018). Macrophage-Membrane-Coated Nanoparticles for Tumor-Targeted Chemotherapy. Nano Lett..

[101] Wang C., Wang S., Chen Y., Zhao J., Han S., Zhao G., Kang J., Liu Y., Wang L., Wang X. (2021). Membrane Nanoparticles Derived from ACE2-Rich Cells Block SARS-CoV-2 Infection. ACS Nano.

[102] Ma J., Xie Y., Teng Z., Jiang L., Liu G. (2025). Engineered cell membrane-based nano therapies fight infectious diseases. J. Control. Release.

[103] Manson J., Kumar D., Meenan B.J., Dixon D. (2011). Polyethylene glycol functionalized gold nanoparticles: The influence of capping density on stability in various media. Gold Bull..

[104] Harrison E., Nicol J.R., Macias–Montero M., Burke G.A., Coulter J.A., Meenan B.J., Dixon D. (2016). A comparison of gold nanoparticle surface co-functionalization approaches using Polyethylene Glycol (PEG) and the effect on stability, non-specific protein adsorption and internalization. Mat. Sci. Eng. C-Mater..

[105] Li X., Huang Y., Jin Q., Ji J. (2021). Mixed-charge modification as a robust method to realize the antiviral ability of gold nanoparticles in a high protein environment. Nanoscale.

[106] Goodman C.M., McCusker C.D., Yilmaz T., Rotello V.M. (2004). Toxicity of Gold Nanoparticles Functionalized with Cationic and Anionic Side Chains. Bioconjug. Chem..

[107] Alkilany A.M., Murphy C.J. (2010). Toxicity and cellular uptake of gold nanoparticles: What we have learned so far?. J. Nanopart. Res..

[108] Budhadev D., Hooper J., Rocha C., Nehlmeier I., Kempf A.M., Hoffmann M., Krüger N., Zhou D., Pöhlmann S., Guo Y. (2023). Polyvalent Nano-Lectin Potently Neutralizes SARS-CoV-2 by Targeting Glycans on the Viral Spike Protein. JACS Au.

[109] Ning X., Budhadev D., Pollastri S., Nehlmeier I., Kempf A., Manfield I., Turnbull W.B., Pöhlmann S., Bernardi A., Li X. (2024). Polyvalent Glycomimetic-Gold Nanoparticles Revealing Critical Roles of Glycan Display on Multivalent Lectin–Glycan Interaction Biophysics and Antiviral Properties. JACS Au.

[110] Budhadev D., Poole E., Nehlmeier I., Liu Y., Hooper J., Kalverda E., Akshath U.S., Hondow N., Turnbull W.B., Pöhlmann S. (2020). Glycan-Gold Nanoparticles as Multifunctional Probes for Multivalent Lectin–Carbohydrate Binding: Implications for Blocking Virus Infection and Nanoparticle Assembly. J. Am. Chem. Soc..

[111] Yang M.-L., Yeh N.-C., Wu C.-L., Shiau A.-L., Chen Y.-H. (2025). Galectin-1-conjugated gold nanoparticles as a multivalent macromolecular platform for broad-spectrum inhibition of influenza virus via glycan recognition. Int. J. Biol. Macromol..

[112] Sergeeva Y., Yeung S.Y., Hix-Janssens T., Sellergren B. (2025). Gold Nanoparticles with Adaptable Self-Assembled Monolayer Shells Allow Multivalent Inhibition and Sensing of Influenza Virus at Ultralow Concentrations. ACS Cent. Sci..

[113] Bhebhe L.M., Kim J., Jones L.M., Super E.H., Jones S.T. (2024). Antiviral mechanism change of poly(styrene sulfonate) through gold nanoparticle coating. Polym. Chem..

[114] Kim J., Yeom M., Lee T., Kim H.-O., Na W., Kang A., Lim J.-W., Park G., Park C., Song D. (2020). Porous gold nanoparticles for attenuating infectivity of influenza A virus. J. Nanobiotechnol..

[115] Elste J., Kumari S., Sharma N., Razo E.P., Azhar E., Gao F., Nunez M.C., Anwar W., Mitchell J.C., Tiwari V. (2023). Plant Cell-Engineered Gold Nanoparticles Conjugated to Quercetin Inhibit SARS-CoV-2 and HSV-1 Entry. Int. J. Mol. Sci..

[116] Ren M., Zhou J., Song Z., Mei H., Zhou M., Fu Z.F., Han H., Zhao L. (2021). Aptamer and RVG functionalized gold nanorods for targeted photothermal therapy of neurotropic virus infection in the mouse brain. Chem. Eng. J..

[117] DeRussy B.M., Aylward M.A., Fan Z., Ray P.C., Tandon R. (2014). Inhibition of cytomegalovirus infection and photothermolysis of infected cells using bioconjugated gold nanoparticles. Sci. Rep..

[118] Bai Y., Zhou Y., Liu H., Fang L., Liang J., Xiao S. (2018). Glutathione-Stabilized Fluorescent Gold Nanoclusters Vary in Their Influences on the Proliferation of Pseudorabies Virus and Porcine Reproductive and Respiratory Syndrome Virus. ACS Appl. Nano Mater..

[119] Feng C., Fang P., Zhou Y., Liu L., Fang L., Xiao S., Liang J. (2018). Different Effects of His-Au NCs and MES-Au NCs on the Propagation of Pseudorabies Virus. Glob. Chall..

[120] De Carli A., Favaro D., Filipponi C., Filippini F., Fonnesu R., Plicanti E., Nottoli S., Barski P., Lindstaedt A., Witt D. (2025). Fighting RNA viruses with a gold nanoparticle Cas13d gene-editing armor. Mol. Ther. Nucl. Acids.

[121] Rafiei S., Rezatofighi* S.E., Ardakani M.R., Rastegarzadeh S. (2016). Gold Nanoparticles Impair Foot-and-Mouth Disease Virus Replication. IEEE Trans. Nanobiosci..

[122] Li C.M., Zheng L.L., Yang X.X., Wan X.Y., Wu W.B., Zhen S.J., Li Y.F., Luo L.F., Huang C.Z. (2016). DNA-AuNP networks on cell membranes as a protective barrier to inhibit viral attachment, entry and budding. Biomaterials.

[123] Li F., Huang Q., Zhou Z., Guan Q., Ye F., Huang B., Guo W., Liang X.-J. (2023). Gold nanoparticles combat enveloped RNA virus by affecting organelle dynamics. Signal Transduct. Target. Ther..

[124] Carja G., Grosu E.F., Petrarean C., Nichita N. (2015). Self-assemblies of plasmonic gold/layered double hydroxides with highly efficient antiviral effect against the hepatitis B virus. Nano Res..

[125] Yang W., Liang H., Ma S., Wang D., Huang J. (2019). Gold nanoparticle based photothermal therapy: Development and application for effective cancer treatment. Sustain. Mater. Technol..

[126] Taylor M.L., Wilson R.E., Amrhein K.D., Huang X. (2022). Gold Nanorod-Assisted Photothermal Therapy and Improvement Strategies. Bioengineering.

[127] Zhou R., Zhang M., Xi J., Li J., Ma R., Ren L., Bai Z., Qi K., Li X. (2022). Gold Nanorods-Based Photothermal Therapy: Interactions Between Biostructure, Nanomaterial, and Near-Infrared Irradiation. Nanoscale Res. Lett..

[128] Wang J., Zhang Y., Jin N., Mao C., Yang M. (2019). Protein-Induced Gold Nanoparticle Assembly for Improving the Photothermal Effect in Cancer Therapy. ACS Appl. Mater. Interfaces.

[129] Nguyen H.A., Darwish S., Pham H.N., Ammar S., Ha-Duong N.-T. (2023). Gold and Iron Oxide Nanoparticle Assemblies on Turnip Yellow Mosaic Virus for In-Solution Photothermal Experiments. Nanomaterials.

[130] Chen J., Li Y., Xiao L., Guo Z., Lu W., Huang J., Li J., Li B., Liu Z. (2025). An Intranasal Nanomedicine Functions as Both Potent Broad-spectrum Viral Inhibitor and Quasi-Vaccine. Adv. Funct. Mater..

[131] Buiel J., Robert J., Mekhjian D., Chauhan D.S., Banquy X. (2025). Photothermal Therapy: From Encouraging Lab Results to Lackluster Clinical Translation. Adv. Ther..

[132] Tao W., Hurst B.L., Shakya A.K., Uddin M.J., Ingrole R.S.J., Hernandez-Sanabria M., Arya R.P., Bimler L., Paust S., Tarbet E.B. (2017). Consensus M2e peptide conjugated to gold nanoparticles confers protection against H1N1, H3N2 and H5N1 influenza A viruses. Antivir. Res..

[133] Tao W., Gill H.S. (2015). M2e-immobilized gold nanoparticles as influenza A vaccine: Role of soluble M2e and longevity of protection. Vaccine.

[134] Bimler L., Song A.Y., Le D.T., Murphy Schafer A., Paust S. (2019). AuNP-M2e + sCpG vaccination of juvenile mice generates lifelong protective immunity to influenza A virus infection. Immun. Ageing.

[135] Wang C., Zhu W., Wang B.Z. (2017). Dual-linker gold nanoparticles as adjuvanting carriers for multivalent display of recombinant influenza hemagglutinin trimers and flagellin improve the immunological responses in vivo and in vitro. Int. J. Nanomed..

[136] Farfán-Castro S., García-Soto M.J., Betancourt-Mendiola L., Cervantes J., Segura R., González-Ortega O., Rosales-Mendoza S. (2024). Synthesis and evaluation of gold nanoparticles conjugated with five antigenic peptides derived from the spike protein of SARS-CoV-2 for vaccine development. Front. Nanotechnol..

[137] Staroverov S.A., Volkov A.A., Mezhenny P.V., Domnitsky I.Y., Fomin A.S., Kozlov S.V., Dykman L.A., Guliy O.I. (2019). Prospects for the use of spherical gold nanoparticles in immunization. Appl. Microbiol. Biot..

[138] Janicka M., Chodkowski M., Osińska A., Bylińska K., Obuch-Woszczatyńska O., Patrycy M., Chodaczek G., Ranoszek-Soliwoda K., Tomaszewska E., Celichowski G. (2025). Adjuvanticity of Tannic Acid-Modified Nanoparticles Improves Effectiveness of the Antiviral Response. Int. J. Nanomed..

[139] Dhandapani S., Ha Y., Wang R., Kwon T.W., Cho I.-H., Kim Y.-J. (2025). Lipid-encapsulated gold nanoparticles: An advanced strategy for attenuating the inflammatory response in SARS-CoV-2 infection. J. Nanobiotechnol..

[140] Kartouzian A., Heiz A., Shameli K., Moeini H. (2025). Polyethylenimine-Conjugated Au-NPs as an Efficient Vehicle for in vitro and in vivo DNA Vaccine Delivery. Int. J. Nanomed..

[141] Lin Z.-Y., Chen Y.-L., Wu C.-L., Chen Y.-H., Chen M.-C. (2026). Augmenting Subunit-Vaccine-Induced Immunity through a Dual Strategy of Gold Nanoparticle Conjugation and Chitosan Microneedle-Mediated Sustained Delivery. ACS Appl. Mater. Interfaces.

